# First Comparative Transcriptomic Analysis of Wild Adult Male and Female *Lutzomyia longipalpis*, Vector of Visceral Leishmaniasis

**DOI:** 10.1371/journal.pone.0058645

**Published:** 2013-03-12

**Authors:** Christina B. McCarthy, María Soledad Santini, Paulo F. P. Pimenta, Luis A. Diambra

**Affiliations:** 1 Centro Regional de Estudios Genómicos, Facultad de Ciencias Exactas, Universidad Nacional de La Plata, Florencio Varela, Buenos Aires, Argentina; 2 Departamento de Informática y Tecnología, Universidad Nacional del Noroeste de la Provincia de Buenos Aires, Pergamino, Buenos Aires, Argentina; 3 Centro Nacional de Diagnóstico e Investigación en Endemoepidemias, Administración Nacional de Laboratorios e Institutos de Salud, Ministerio de Salud, Buenos Aires, Argentina; 4 Laboratory of Medical Entomology, Centro de Pesquisas René Rachou, Fundação Oswaldo Cruz – FIOCRUZ, Belo Horizonte, Minas Gerais, Brazil; Washington State University, United States of America

## Abstract

Leishmaniasis is a vector-borne disease with a complex epidemiology and ecology. Visceral leishmaniasis (VL) is its most severe clinical form as it results in death if not treated. In Latin America VL is caused by the protist parasite *Leishmania infantum* (syn. *chagasi*) and transmitted by *Lutzomyia longipalpis*. This phlebotomine sand fly is only found in the New World, from Mexico to Argentina. However, due to deforestation, migration and urbanisation, among others, VL in Latin America is undergoing an evident geographic expansion as well as dramatic changes in its transmission patterns. In this context, the first VL outbreak was recently reported in Argentina, which has already caused 7 deaths and 83 reported cases.

Insect vector transcriptomic analyses enable the identification of molecules involved in the insect's biology and vector-parasite interaction. Previous studies on laboratory reared *Lu. longipalpis* have provided a descriptive repertoire of gene expression in the whole insect, midgut, salivary gland and male reproductive organs. Nevertheless, the study of wild specimens would contribute a unique insight into the development of novel bioinsecticides. Given the recent VL outbreak in Argentina and the compelling need to develop appropriate control strategies, this study focused on wild male and female *Lu. longipalpis* from an Argentine endemic (Posadas, Misiones) and a Brazilian non-endemic (Lapinha Cave, Minas Gerais) VL location. In this study, total RNA was extracted from the sand flies, submitted to sequence independent amplification and high-throughput pyrosequencing. This is the first time an unbiased and comprehensive transcriptomic approach has been used to analyse an infectious disease vector in its natural environment. Transcripts identified in the sand flies showed characteristic profiles which correlated with the environment of origin and with taxa previously identified in these same specimens. Among these, various genes represented putative targets for vector control via RNA interference (RNAi).

## Introduction

Leishmaniasis, a vector-borne neglected infectious disease of worldwide incidence, comprises two major diseases: visceral leishmaniasis (VL), which is fatal if untreated, and the cutaneous form (CL), which can heal spontaneously but leaves disfiguring scars [1]. Leishmaniasis is classified as one of the ‘‘most neglected diseases’’ [2] due to the limited resources invested in its diagnosis, treatment and control and its strong association with poverty [3]. Moreover, the estimated burden for this disease places it second in mortality and fourth in morbidity among all tropical diseases [4]. Like many other neglected tropical diseases, leishmaniasis occurs in a focal distribution and in remote locations, which makes extrapolation from official data sources difficult [5]. Furthermore, since VL results in death if not treated, the majority of deaths caused by leishmaniasis go unrecognized and, even with access to treatment, VL may result in case-fatality rates of 10–20% [6], [7], [8], the reported leishmaniasis case figures are widely acknowledged to represent gross underestimates of the true burden [9]. Given leishmaniasis is strongly associated with poverty, this burden falls disproportionately on the world's poorest. Within endemic areas, infection risk is increased by poor housing conditions and environmental sanitation, lack of personal protective measures, and economically driven migration, which brings non-immune hosts into contact with infected sand flies. Furthermore, inadequate access to healthcare causes delays in appropriate diagnosis and treatment and accentuates leishmaniasis morbidity and mortality [3].

Leishmaniasis is transmitted through the bite of two phlebotomine sand fly genera, *Phlebotomus* in the Old World and *Lutzomyia* in the New World [10]. In Latin America VL is caused by *Leishmania infantum* (syn. *chagasi*) and transmitted by *Lutzomyia longipalpis*. This phlebotomine sand fly is only found in the New World, with a wide distribution from Mexico to Argentina [11]. The geographical distribution of leishmaniasis has undoubtedly expanded and is now being reported in areas that were previously non-endemic. The worldwide phenomenon of urbanisation, closely related to the sharp increase in migration, is one of the major risk factors that is making leishmaniasis a growing public health concern for many countries around the world [12], and Argentina is not an exception. The first VL case was described in 1926 in the Chaco region and, between 1926 and 1989, 14 cases which were not attributed to *Le. infantum* were reported within the endemic CL area in Salta province. Moreover, *Lu. longipalpis* was reported on two isolated occasions (in 1953 and 2000) and these reports were not associated with VL [13]. Nevertheless, this situation has changed dramatically and an autochthonous urban focus was recently reported in the city of Posadas (Misiones province) [14], with case numbers increasing every year. From 2006 to 2011 the morbidity and mortality toll of this disease amounted to 83 human cases (35% corresponding to children under ten years of age), 7 deaths and more than 7,000 infected dogs (National Health Surveillance System, Epidemiology Bureau, National Ministry of Health, Argentina). Moreover, it is suspected that VL is underreported (2 to 4-fold) due to its recent introduction in the country, a lack of awareness in health workers and an increasing number of infected dogs [15].

Transcriptomics is a powerful tool for the identification of molecules expressed in a whole organism or in a particular tissue. Previous *Lu. longipalpis* transcriptomic studies focused on the whole body [16], midgut [17], [18], salivary gland [19], [20] and, recently, male reproductive organs [21]. The study by Dillon *et al.* [16], in which transcripts were generated using whole *Lu. longipalpis*, combining unfed, blood-fed or infected (with *Le. infantum*, *Le. mexicana* or bacteria) sand flies, provided a global descriptive repertoire of sand fly molecules. Two *Lu. longipalpis* midgut transcriptome analyses followed [17], [18] which used vector strains from different geographical regions in Brazil and different parasite strains. These studies offered a specific characterisation of midgut molecules and revealed the ability of *Leishmania* parasites to modulate vector midgut transcripts. A recent comparative salivary gland transcriptomic study identified families of salivary proteins common to all the sand flies studied, proteins that were genus specific and proteins that were species specific [20]. The only other transcriptomic study to date that has analysed adult male *Lu. longipalpis*, reported the transcriptome of *Lu. longipalpis* male reproductive organs and the identification of a number of putative male reproductive gland proteins [21]. These studies have comprised a first and necessary step to identify molecules involved in sand fly-*Leishmania* interactions.

Nonetheless, transcriptomic analyses of infectious disease vectors in their natural environment represent an invaluable tool, not only to provide a novel insight into their biology, but also to identify genes expressed ‘in the wild’ to be targeted for vector control. In this context and given the recent VL outbreak in Argentina, this study used unbiased high-throughput pyrosequencing technology to compare the transcriptomes of wild male and female adult *Lu. longipalpis* from endemic (Posadas, Misiones) and non-endemic (Lapinha Cave, Minas Gerais) VL locations in Argentina and Brazil, respectively. A comprehensive analysis was performed which integrated gene expression data with environmental information and taxa previously associated with these same sand flies [22].

## Methods

### Ethics Statement

This study was carried out in strict accordance with the recommendations in the Manual for the Use of Animals/FIOCRUZ (Manual de Utilização de Animais/ FIOCRUZ) of Fundação Oswaldo Cruz, FIOCRUZ, Ministry of Health of Brazil (National decree Nr 3,179). The protocol was approved by the Ethics Committee for the Use of Animals of the Fundação Oswaldo Cruz - FIOCRUZ, Ministry of Health of Brazil (Nr 242/99).

### Field sampling and specimen preparation

*Lu. longipalpis* specimens from the non-endemic VL location were obtained from Lapinha Cave (Minas Gerais, Brazil), located in the Sumidouro State Park. Sand flies from this location were chosen as reference because they have been extensively studied. *Lu. longipalpis* specimens from the endemic VL location were obtained from Posadas (Misiones, Argentina), where they occur in high density. Moreover, a previous study using polymorphic markers showed that the *Lu. longipalpis* population from Posadas (Argentina) is significantly differentiated from Brazilian populations, including the Lapinha population [23]. Captures were made using CDC light traps [24] on the 15th and 26th of May 2009 in the Lapinha Cave and in Posadas, respectively. In the Lapinha Cave (S19 33 42.42 W43 57 34.96) the trap was left 50–80 cm from ground level and a chicken was kept to attract the sand flies and as a source of food. In Posadas, the trap was installed in the peridomicile of a worst-case scenario homestead (domestic animals, dense vegetation, nearby spring) (S27 23.266 W55 53.403). Detailed descriptions of the capture sites can be found in [22].

Sand flies were transported alive in a nylon cage to the corresponding laboratories in Belo Horizonte (Minas Gerais) and Posadas (Misiones), where they were killed at low temperature, identified and separated according to sex, and stored alternatively in Tri-Reagent (Molecular Research Center Inc., Cincinnati, OH) or RNAlater® (Qiagen). A total of four groups of 100 sand flies each, two per location, were separated and named according to: SS1, females from the Endemic VL location (EVL females); SS2, EVL males; PP1, females from the Non-Endemic VL location (NEVL females); and PP2, NEVL males.

### Sample preparation

Individual samples were ground in Tri-Reagent (Molecular Research Center Inc., Cincinnati, OH) with a Teflon pestle and total RNA was immediately extracted, according to the manufacturer's instructions. Total RNA was amplified using a modified sequence-independent amplification protocol [25], as described in [22]. Briefly, M-MuLV Reverse Transcriptase (Fermentas, Vilnius, Lithuania) was used for first-strand reverse transcription which was initiated with a random octamer linked to a specific primer sequence (5′-GTT TCC CAG TAG GTC TCN NNN NNN N-3′). cDNA was then amplified with the Expand Long Template PCR System (Roche) using a 1∶9 mixture of the above primer and a primer targeting the specific primer sequence (5′-CGC CGT TTC CCA GTA GGT CTC-3′) [26]. The following profile was used: initial denaturation cycle at 94°C for 2 minutes; five low stringency cycles with denaturation at 94°C for 30 seconds, 25°C for 30 seconds and 68°C for 6 minutes, were followed by 30 cycles at 94°C for 30 seconds, 55°C for 30 seconds and 68°C for 6 minutes, and a final extension cycle at 68°C for 5 minutes. Pooled samples were submitted for high-throughput pyrosequencing (Macrogen Inc., Korea).

### Sequence accession numbers

Reads were submitted to the NCBI Sequence Read Archive (SRA) (submission SRA026595) under accessions SRX031655 (adult female *Lu. longipalpis* from the endemic VL location Posadas, Misiones, Argentina; SS1), SRX031656 (adult male *Lu. longipalpis* from the endemic VL location Posadas, Misiones, Argentina; SS2), SRX036778 (adult female *Lu. longipalpis* from the non-endemic VL location Lapinha Cave, Minas Gerais, Brazil; PP1) and SRX036754 (adult male *Lu. longipalpis* from the non-endemic VL location Lapinha Cave, Minas Gerais, Brazil; PP2).

### Sequence analysis

Reads ranged in size from approximately 100 to 1200 base pairs (bp) (350 bp average). Raw sequence reads were trimmed to remove sequences derived from the amplification primer using a custom application written in Mathematica (Wolfram Mathematica 7; available upon request). With the purpose of reducing database search efforts and improving the homology detection sensitivity [27], CD-HIT [28] was used to generate non-redundant nucleotide datasets. Nevertheless, as redundancy represented less than 1% (at 95% identity) in every case (data not shown), using the non-redundant nucleotide datasets would not have reduced database search efforts neither improved the performance of other sequence analyses. Consequently, trimmed singlet reads were used for the database searches. A preliminary version of the *Lu. longipalpis* genome (Llon v1.0 contigs, here referred to as Llon_contigs) was downloaded from the Baylor College of Medicine Human Genome Sequencing Center (BCM-HGSC) website (http://www.hgsc.bcm.tmc.edu). Additionally, four databases were downloaded locally: non-redundant nucleotide sequences (nt), 16S ribosomal RNA sequences (Bacteria and Archaea) (16S), non-human, non-mouse ESTs (est-others), and non-redundant protein sequences (nr) NCBI databases, last modified on 08/06/12, 12/06/12, 14/06/12 and 08/06/12, respectively (ftp://ftp.ncbi.nlm.nih.gov/blast/db/); and UniProt Knowledgebase (uniprotKB), consisting of the Swiss-Prot Protein Knowledgebase (fully annotated curated entries) and the TrEMBL Protein Sequence Database (computer-generated entries enriched with automated classification and annotation) (uniprotKB  =  uniprot_sprot + uniprot_trembl), last modified on 13/06/12.

**Figure 1:**
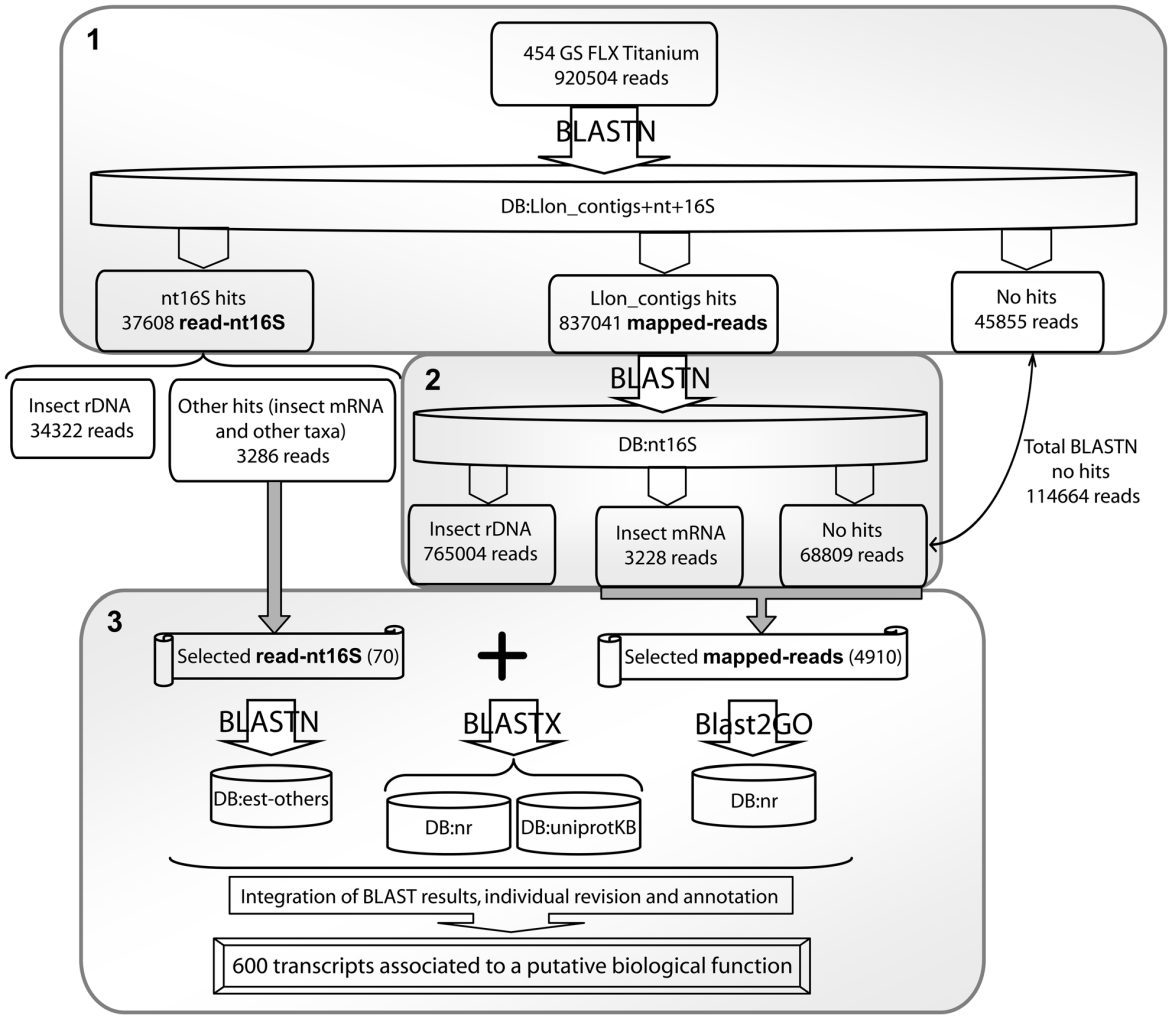
Sequence analysis workflow. This figure shows an overview of the rationale supporting the analysis of the reads (transcripts) obtained in this study and summarises the different steps that were followed. 1) Indicates the first stage of analysis, in which all the reads were blasted against DB:Llon_contigs+nt+16S. Results were classified in three datasets: reads that mapped to Llon_contigs by sequence homology (mapped-reads); reads that showed homology to nt16S (read-nt16S); and reads that returned no significant hits (no hits) (see text for details). 2) Indicates the second stage of analysis, in which the mapped-reads dataset was blasted against DB:nt16S (see text for details). 3) Indicates the third stage of analysis, in which selected reads (mapped-reads and read-nt16S) were separately blasted against three databases (est-others, nr and uniprotKB) and annotated using Blast2GO (see text for details). The ‘selected mapped-reads (4910)’ excluded mapped-reads 15379, 23694, 25834, 27903, 27904 and 9281, which showed homology to insect rDNA after BLASTN against DB:nt16S, and contig 31202, with unknown function after BLASTN against DB:nt16S (see text for details). The ‘selected read-nt16S (70)’ included read-nt16S that did not show homology to either insect rDNA or taxa other than insects (see text for details).

In a first stage, sequences from Llon_contigs, nt and 16S were combined in a single database (DB:Llon_contigs+nt+16S) using the appropriate BLAST+ applications (ftp://ftp.ncbi.nlm.nih.gov/blast/executables/blast/LATEST/). Trimmed singlet reads were compared to this combined database using BLASTN (nucleotide homology) [29], with a 1e-50 cutoff E-value (Figure 1). Reads which mapped to Llon_contigs by sequence homology (here referred to as mapped-reads) were then separated from those that showed homology to nt16S (here referred to as read-nt16S), and an accurate record was kept of which Llon_contig each mapped-read showed homology to, using custom applications written in Mathematica (Wolfram Mathematica 7; available upon request) (Figure 1). To simplify the interpretation of data in this study, each mapped-read will be referred to according to the Llon_contig it showed homology to. For example, mapped-reads that showed homology to Llon_contig 15380 will be referred to as mapped-reads 15380. To further clarify the use of terminology in this study, whenever ‘mapped-reads’ and/or ‘read-nt16S’ and/or ‘reads’ and/or ‘transcripts’ are mentioned, we are referring to transcripts generated in this study, and whenever ‘Llon_contigs’ are mentioned, we are referring to the *Lu. longipalpis* genome sequence data (downloaded from http://www.hgsc.bcm.tmc.edu). Results from the first stage of analysis were organised in three datasets: mapped-reads (reads that mapped to Llon_contigs by sequence homology), read-nt16S (reads that showed homology to nt16S) and no hits (reads that returned no significant hits). Results from the read-nt16S dataset were then classified using custom applications written in Mathematica (Wolfram Mathematica 7; available upon request) and those read-nt16S that did not show homology to insect rDNA or taxa other than insects, were selected and separated from the rest to be further analysed (Figure 1).

In a second stage, database sequences from nt and 16S were combined in a single database (DB:nt16S) using the appropriate BLAST+ applications (ftp://ftp.ncbi.nlm.nih.gov/blast/executables/blast/LATEST/), and the mapped-reads dataset was compared to DB:nt16S using BLASTN [29] with a 1e-50 cutoff E-value. After analysing the results of this homology search, it was observed that mapped-reads which in the first stage showed homology to Llon_contigs 15379, 23694, 25834, 27903, 27904 and 9281, after BLASTN against DB:nt16S, overall showed homology to insect rDNA (see Results; these were the only Llon_contigs that were partially annotated after blasting their corresponding mapped-reads against DB:nt16S, i.e. the second stage of analysis), and mapped-reads that in the first stage showed homology to Llon_contig 31202, after BLASTN against DB:nt16S, overall showed no significant hits. Therefore, these mapped-reads were excluded from the third stage of analysis (Figure 1).

In the third stage of analysis, all mapped-reads excluding the aforementioned ones (a total of 4910 mapped-reads considering all four samples) and the selected read-nt16S (i.e. those that did not show homology to insect rDNA or taxa other than insects, see above), were then compared separately to three databases: DB:est-others using BLASTN [29] with a 1e-50 cutoff E-value, and DB:nr and DB:uniprotKB using BLASTX [29] with a 1e-6 cutoff E-value (these homology search results are provided as supplementary material: Tables S13–S28 for the selected mapped-reads and Tables S9–S12 second sheet for the selected read-nt16S); these mapped-reads and read-nt16S were also analysed and annotated using Blast2GO [30] (Figure 1 and Tables S1, S9–S12). Hits from all four databases and Blast2GO results were compared and individually revised and confirmed, and only those which showed unequivocal results were included in the final analysis (Tables S1 and S9–S12). Putative functional assignment and categorisation for each selected mapped-read and read-nt16S was individually and manually revised and assigned on the basis of BLASTX results (mainly DB:uniprotKB) and Blast2GO annotation.

This analysis was performed separately for each sample.

Fisher's Exact Test (FET) [31] (p<0.05) was used to establish the significance of reads in the different samples using custom applications written in Mathematica (Wolfram Mathematica 7; available upon request). The statistical analysis of reads associated with the different functional biological categories (see below), was performed on the basis of the number of selected transcripts that were submitted to the third stage of analysis (i.e. compared separately to DB:est-others, DB:nr and DB:uniprotKB and analysed and annotated using Blast2GO) for each sample and the total number of transcripts that were assigned a given putative biological function for each sample. The statistical analysis of mapped-reads was performed on the basis of the total number of mapped-reads for each sample and the number of mapped-reads that showed homology to a given Llon_contig. This analysis was limited to those samples in which the Llon_contigs showed consistent mapped-read homology search results (i.e. homology to the same gene or to members of the same multigene family).

## Results

### General considerations

A total of 920,504 reads were obtained from all four cDNA libraries; 479,781 of these (52.12%) corresponded to cDNA from females and 440,723 (47.88%) to cDNA from males. Due to the fact that total RNA was amplified using a modified sequence-independent amplification protocol [25] and, for this, first-strand reverse transcription was initiated with a random octamer linked to a specific primer sequence, an unbiased random collection of transcripts was obtained for each sample.

Figure 1 shows an overview of the rationale supporting the sequence analysis workflow in this study, summarising the different steps that were followed. From the first stage of analysis (i.e. total reads blasted against DB:Llon_contigs+nt+16S), a total of 837,041 reads (90.93%) mapped to Llon_contigs by sequence homology (mapped-reads), 37,608 reads (4.09%) showed homology to nt16S (read-nt16S) and 45,855 reads (4.98%) returned no significant hits (Figure 1) (homology search results not shown). Of the 37,608 read-nt16S, 34,322 reads corresponded to insect rDNA (mostly *Lu. longipalpis* 28S ribosomal RNA gene, accessions FJ040565.1 and AY349492.1) and the rest (3286 reads) to other taxa and insect mRNAs (Figure 1) (homology search results not shown).

From the second stage of analysis, after blasting the 837,041 mapped-reads against DB:nt16S, 765,004 reads (91.39%) showed homology to insect rDNA (overall homology to *Lu. longipalpis* 28S ribosomal rDNA, accession FJ040565.1, *Lu. longipalpis* 18S rRNA gene, accession AJ244429.1 and *Ph. duboscqi* 28S ribosomal RNA gene, accession FJ040566.1), 3,228 reads (0.39%) showed homology to insect mRNA (mainly *Lu. longipalpis*) and 68,809 reads (8.22%) showed no significant hits (Figure 1) (homology search results are provided as supplementary material only for the selected mapped-reads, see below; Tables S13, S17, S21 and S25).

The number of reads that showed no significant hits after the first and second BLASTN homology searches against DB:Llon_contigs+nt+16S and DB:nt16S, was 45855 and 68809, respectively (a total of 114,664, 12.46%) (Figure 1).

After the second stage of analysis, mapped-reads that showed homology to insect rDNA included: mapped-reads 15379 (overall homology to *Lu. longipalpis* 28S ribosomal rDNA, accession FJ040565.1), 23694 (overall homology to FJ040565.1), 25834 (overall homology to FJ040565.1), 27903 (overall homology to *Lu. longipalpis* 18S rRNA gene, accession AJ244429.1), 27904 (overall homology to *Ph. duboscqi* 28S ribosomal RNA gene, accession FJ040566.1) and 9281 (overall homology to FJ040565.1) (homology search results not shown). Furthermore, mapped-reads 31202 were abundant in all samples and, after being blasted against DB:nt16S, overall showed no significant hits. Thus, as mapped-reads 15379, 23694, 25834, 27903, 27904 and 9281 overall showed homology to insect rDNA and mapped-reads 31202 predominantly returned no significant hits, all mapped-reads except the aforementioned ones were submitted to the next stage of analysis.

In the third stage of analysis, selected mapped-reads (which, as mentioned previously, excluded mapped-reads 15379, 23694, 25834, 27903, 27904, 9281 and 31202) and read-nt16S (those that did not show homology to insect rDNA or taxa other than insects), were then blasted separately against DB:est-others and protein databases DB:nr and DB:uniprotKB (homology search results of the selected reads for the different samples are provided as supplementary material: Tables S14–S16, S18–S20, S22–S24 and S26–S28 for selected mapped-reads, and Tables S9–S12 second sheet for selected read-nt16S). These selected mapped-reads and read-nt16S were also analysed and annotated using Blast2GO. Putative gene function for each selected mapped-read and read-nt16S was individually and manually revised and assigned on the basis of BLASTX results (mainly DB:uniprotKB) and Blast2GO annotation. Reads with homology to unknown conserved proteins and unknown proteins that could not be associated with a putative function were excluded from the final analysis. For SS1 (EVL females), 620 transcripts (617 mapped-reads and 3 read-nt16S; Tables S14–S16 and S9 second sheet, respectively) were further blasted against DB:est-others, DB:nr and DB:uniprotKB. Of these, 92 transcripts (14.8%) were assigned a putative biological function (Table S9). For SS2 (EVL males), 1,037 transcripts (1,030 mapped-reads and 7 read-nt16S; Tables S18–20 and S10 second sheet, respectively) were further blasted against DB:est-others, DB:nr and DB:uniprotKB. Of these, 86 transcripts (8.3%) were assigned a putative biological function (Table S10). For PP1 (NEVL females), 1,988 transcripts (1,977 mapped-reads and 11 read-nt16S; Tables S22–24 and S11 second sheet, respectively) were further blasted against DB:est-others, DB:nr and DB:uniprotKB. Of these, 331 transcripts (16.6%) were assigned a putative biological function (Table S11). For PP2 (NEVL males), 1,335 transcripts (1,286 mapped-reads and 49 read-nt16S; Tables S26–28 and S12 second sheet, respectively) were further blasted against DB:est-others, DB:nr and DB:uniprotKB. Of these, 91 transcripts (6.8%) were assigned a putative biological function (Table S12). Considering all four samples, a total of 4,910 mapped-reads and 70 read-nt16S were further blasted against DB:est-others, DB:nr and DB:uniprotKB and, of these, 600 transcripts were assigned a putative biological function (12.2%) (Figure 1 and Tables S9–S12).

We recently published the results of a metagenomic analysis of these same samples [22] using a different approach to the one presented here, since the objective of that study was to survey taxa associated with this infectious disease vector. Our previous analysis revealed the presence of sequences from bacteria, fungi, protist parasites, plants and metazoans. Notwithstanding the differences in both approaches, overall results from both studies were consistent and complementary and enabled a comprehensive analysis of these sand flies as environmental samples.

### Putative biological functions assigned to transcripts (mapped-reads and read-nt16S) obtained in this study

The previously mentioned procedure for the assignment of putative gene function for each selected mapped-read and read-nt16S on the basis of BLASTX results and Blast2GO annotation, enabled the association of putative general biological functions to a total of 600 transcripts, considering all four samples. In the following sections the number of transcripts assigned to the different function categories in each library and an abbreviated list of the genes identified in each functional class, are mentioned.

### Transcripts associated with environmental stress, immunity and resistance to xenobiotics

**Figure 2:**
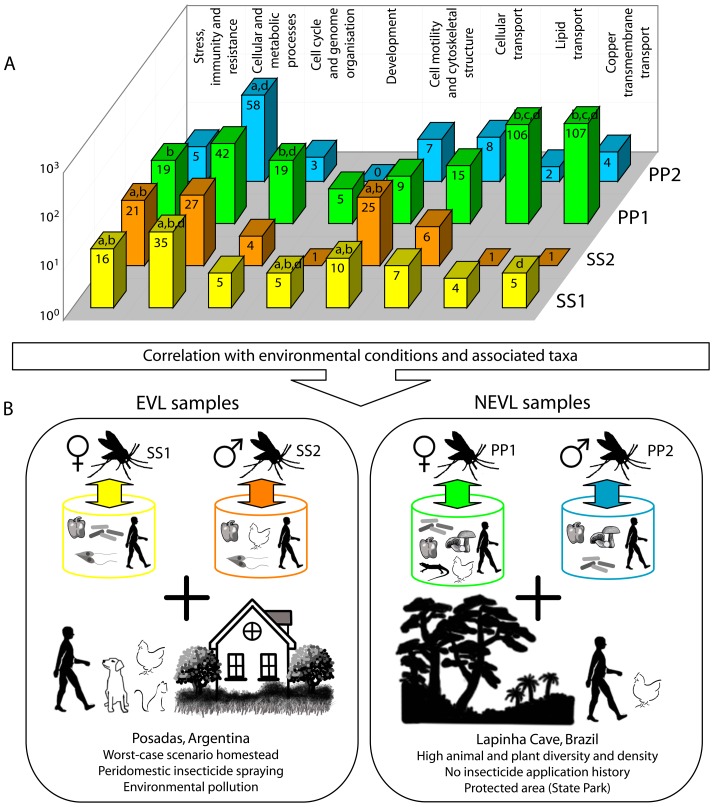
Sand flies as environmental samples: expression profiles in EVL and NEVL sand flies correlated with environmental conditions and taxa previously identified in these samples. This figure integrates data from the function categories identified in these wild EVL and NEVL sand flies with sampling site characteristics and taxa previously found in these same samples [22]. Figures are only schematic and not an exact representation of either the sampling sites, phlebotomine sand flies or identified taxa. A) Shows the function categories that the transcripts were assigned to in all the samples and the number of transcripts assigned to each function category for each sample. Values are expressed on a logarithmic scale and indicated for each sample on the corresponding bar. Significant differences in the number of transcripts in each category between samples (Fisher's Exact Test; p<0.05) are indicated as: a, significantly overrepresented with respect to PP1; b, significantly overrepresented with respect to PP2; c, significantly overrepresented with respect to SS1; and d, significantly overrepresented with respect to SS2. B) The top part shows a schematic of the sandflies (female or male) from both locations and of the taxa we previously identified in all four samples [22]. Barrels group the taxa found in each sample. Previously identified taxa in SS1: bacteria, protists, metazoans (human) and plants; SS2: protists, metazoans (human and chicken) and plants; PP1: bacteria, fungi, metazoans (human, chicken and lizard) and plants; and PP2: bacteria, fungi, metazoans (human) and plants. Taxa are represented schematically and the particular species identified for each taxonomical group are not shown, except in the case of metazoans. The bottom part shows the most significant ecological characteristics of both capture site locations in Argentina and Brazil, Posadas and Lapinha Cave, respectively. Only those animal species confirmed in the sampling sites in both locations at the time of sampling were represented schematically. EVL sampling site (Posadas, Argentina): human, dog, cat and chicken; NEVL sampling site (Lapinha Cave, Brazil): human and chicken. SS1 (indicated in yellow): EVL adult female *Lu. longipalpis* (Posadas, Argentina); SS2 (indicated in orange): EVL adult male *Lu. longipalpis* (Posadas, Argentina); PP1 (indicated in green): NEVL adult female *Lu. longipalpis* (Lapinha Cave, Brazil); PP2 (indicated in pale blue): NEVL adult male *Lu. longipalpis* (Lapinha Cave, Brazil).

A total of 61 transcripts (mapped-reads and read-nt16S) were associated with responses to environmental stress, immunity and resistance to xenobiotics. Of these, 16 corresponded to EVL females (SS1; Figure 2A and Table S9), 21 to EVL males (SS2; Figure 2A and Table S10), 19 to NEVL females (PP1; Figure 2A and Table S11) and 5 to NEVL males (PP2; Figure 2A and Table S12). Transcripts in this category were significantly overrepresented in EVL samples with respect to NEVL samples (Figure 2A). Whereas there was no significant difference between SS1 and SS2, these transcripts were significantly overrepresented in PP1 with respect to PP2 (Figure 2A).

Among others, these transcripts showed homology to heat shock proteins (HSP), catalase, cytochrome p450, mitogen-activated protein kinases (MAPK), autophagy related proteins, multidrug resistance-associated proteins (ATP-binding cassette transporters), senescence-associated proteins, serine protease inhibitors, proteins associated with arsenite transport, DNA mismatch repair proteins, proteins associated with sensory perception of chemical stimuli and immunoglobulin I-set (Ig_I-set) proteins (Tables S9–S12).

### Transcripts associated with cellular and metabolic processes

A total of 162 transcripts (mapped-reads and read-nt16S) were associated with cellular and metabolic processes. Of these, 35 corresponded to EVL females (SS1; Figure 2A and Table S9), 27 to EVL males (SS2; Figure 2A and Table S10), 42 to NEVL females (PP1; Figure 2A and Table S11) and 58 to NEVL males (PP2; Figure 2A and Table S12). Transcripts in this category were significantly overrepresented in SS1 with respect to all the other samples (Figure 2A). There was no significant difference between SS2 and PP1 but both these samples were significantly underrepresented with respect to PP2 (Figure 2A).

Among others, these transcripts were associated with aerobic respiration (cytochrome oxidase subunit I), oxidation-reduction process (short-chain dehydrogenases), protein metabolic process (glutamate semialdehyde dehydrogenase, ubiquinol-cytochrome c reductase complex core protein, oligosaccharyl transferase, pyrroline-5-carboxylate reductase), lipid metabolic process (fatty acid synthase S-acetyltransferase, neural stem cell-derived dendrite regulator/triglyceride lipase activity), transcription (transcription factor E2f, forkhead box transcription factor, UCR-motif DNA binding protein) and translation (elongation factor, ribosomal protein L39) (Tables S9–S12).

### Transcripts associated with cell cycle and genome organisation

A total of 31 transcripts (mapped-reads) were associated with cell cycle and genome organisation. Of these, 5 corresponded to EVL females (SS1; Figure 2A and Table S9), 4 to EVL males (SS2; Figure 2A and Table S10), 19 to NEVL females (PP1; Figure 2A and Table S11) and 3 to NEVL males (PP2; Figure 2A and Table S12). Transcripts in this category were significantly overrepresented in NEVL females with respect to males from both locations. However, there was no significant difference between females, between males, nor between SS1 and males from both locations (Figure 2A).

Among others, these transcripts showed homology to cyclin, mitotic checkpoint protein MAD1, Kakapo, Ras suppressor protein and chromodomain-helicase-DNA-binding protein 1 (Tables S9–S12).

### Transcripts associated with development

A total of 11 transcripts (mapped-reads) were associated with development. Of these, 5 corresponded to EVL females (SS1; Figure 2A and Table S9), 1 to EVL males (SS2; Figure 2A and Table S10), 5 to NEVL females (PP1; Figure 2A and Table S11) and none were identified in NEVL males (PP2; Figure 2A and Table S12). Transcripts in this category were significantly overrepresented in SS1 with respect to all the other samples, but there was no significant difference between PP1 and males from both locations nor between SS2 and PP2 (Figure 2A).

Among others, these transcripts showed homology to maternal protein exuperantia, Bicaudal C and D, Z band alternatively spliced PDZ-motif protein 66, Klarsicht protein and promyelocytic leukemia zinc finger protein (Tables S9–S11).

### Transcripts associated with cellular transport

A total of 36 transcripts (mapped-reads) were associated with cellular transport. Of these, 7 corresponded to EVL females (SS1; Figure 2A and Table S9), 6 to EVL males (SS2; Figure 2A and Table S10), 15 to NEVL females (PP1; Figure 2A and Table S11) and 8 to NEVL males (PP2; Figure 2A and Table S12). There was no significant difference in the number of transcripts between samples (Figure 2A).

Among others, these transcripts showed homology to Ctl2 (choline transporter-like 2), voltage-gated sodium channel Nav1 alpha subunit, sodium/solute symporter and uncharacterised proteins associated with cation:chloride symporter activity and transmembrane transport (Tables S9–S12).

### Transcripts associated with lipid transport and copper transmembrane transport

A total of 113 and 117 transcripts (mapped-reads) were associated with lipid transport and copper transmembrane transport, respectively. Of those associated with lipid transport, the vast majority (106) were identified in NEVL females (PP1; Figure 2A and Table S11), 4 were found in EVL females (SS1; Figure 2A and Table S9), 1 was found in EVL males (SS2; Figure 2A and Table S10) and 2 were found in NEVL males (PP2; Figure 2A and Table S12). Of those transcripts associated with copper transmembrane transport, 107 were found in NEVL females (PP1; Figure 2A and Table S11), 5 in EVL females (SS1; Figure 2A and Table S9), 1 in EVL males (SS2; Figure 2A and Table S10) and 4 in NEVL males (PP2; Figure 2A and Table S12). Transcripts related with lipid transport were clearly overrepresented in PP1 with respect to the rest of the samples. Between the latter, there was no significant difference in expression (Figure 2A). Transcripts related to copper transmembrane transport were also significantly overrepresented in PP1 with respect to the rest of the samples (Figure 2A). However, these transcripts were significantly overrepresented in SS1 with respect to SS2, although there was no significant difference between SS1 and PP2 nor between SS2 and PP2 (Figure 2A).

Transcripts associated with lipid transport showed homology to apolipophorins, cholesterol transport proteins, oxysterol-binding proteins and vitellogenin (Vg) (Tables S9–S12).

Transcripts associated with copper transmembrane transport showed homology to high-affinity copper uptake proteins (Ctr1) (Tables S9–S12).

### Transcripts associated with cell motility and cytoskeletal structure

A total of 51 transcripts (mapped-reads) were associated with cell motility and cytoskeletal structure. Of these, 10 corresponded to EVL females (SS1; Figure 2A and Table S9), 25 to EVL males (SS2; Figure 2A and Table S10), 9 to NEVL females (PP1; Figure 2A and Table S11) and 7 to NEVL males (PP2; Figure 2A and Table S12). These transcripts were significantly overrepresented in SS1 and SS2 with respect to NEVL samples (Figure 2A). Nevertheless, there was no significant difference between SS1 and SS2 nor between PP1 and PP2 (Figure 2A).

Among others, transcripts in this category showed homology to actin, myosin, spectrin, nesprin and titin (Tables S9–S12).

### Analysis of the Llon_contigs reads mapped to by sequence homology

As mentioned previously, total reads were initially blasted against DB:Llon_contigs+nt+16S and the results from this homology search were then classified in three datasets. The first dataset comprised reads that mapped to Llon_contigs by sequence homology (90.93% mapped-reads); the second, reads that showed homology to nt16S (4.09% read-nt16S); and, the third, reads that returned no significant hits (4.98% no hits) (Figure 1). Furthermore, for the mapped-reads dataset, a careful record was kept of which Llon_contig each mapped-read showed homology to. Due to the fact that reads generated in this study were obtained from *Lu. longipalpis* total RNA and that a stringent cutoff E-value was used for the homology searches (1e-50), reads in the mapped-reads dataset (i.e. that mapped to Llon_contigs by sequence homology) were considered unequivocally as *Lu. longipalpis* sequences.

The *Lu. longipalpis* sequence data (downloaded from http://www.hgsc.bcm.tmc.edu) is preliminary and unannotated and was released prior to project completion as a public service to enable the search for genes or functions. In this context, the purpose for keeping an accurate record of which Llon_contig each mapped-read showed homology to, was to contribute towards the partial *in silico* annotation of the Llon_contigs identified by sequence homology (a total of 654 considering all four samples), via subsequent BLAST analyses of the corresponding mapped-reads. Altogether, considering the totality of the mapped-reads, the mentioned rationale supporting the analysis of *Lu. longipalpis* transcripts from this study, enabled the partial *in silico* annotation of almost a third of the Llon_contigs which were identified by sequence homology (176 Llon_contigs, 26.9%) (Table S1 first sheet).

In the case of SS1 (EVL females), a total of 213 Llon_contigs were identified by sequence homology (Table S2 second sheet) and 75 of these (35.2%) were partially annotated via *in silico* sequence analysis of the corresponding mapped-reads (Table S2). For SS2 (EVL males), a total of 179 Llon_contigs were identified by sequence homology (Table S3 second sheet) and 53 of these (29.6%) were partially annotated via *in silico* sequence analysis of the corresponding mapped-reads (Table S3). In the case of PP1 (NEVL females), a total of 315 Llon_contigs were identified by sequence homology (Table S4 second sheet) and 63 of these (20%) were partially annotated via *in silico* sequence analysis of the corresponding mapped-reads (Table S4). For PP2 (NEVL males), a total of 88 Llon_contigs were identified by sequence homology (Table S5 second sheet) and 32 of these (36.4%) were partially annotated via *in silico* sequence analysis of the corresponding mapped-reads (Table S5).

Of the total 654 Llon_contigs that were identified by sequence homology, considering all four samples (data not shown), various were found in more than one library (Table S1). Fifteen Llon_contigs were identified in all four samples and 11 of these were partially annotated via *in silico* sequence analysis of the corresponding mapped-reads: Llon_contig 15380 (stress, immunity and resistance only in PP2; see Discussion); Llon_contig 10948 (cellular transport only in SS1, PP1 and PP2; see Discussion); Llon_contig 5231 (lipid transport only in SS1, SS2 and PP2; see Discussion); Llon_contig 32328 (cell motility and cytoskeletal structure in all four libraries; see Discussion); Llon_contig 33875 (cell cycle in all four libraries; see Discussion); and Llon_contigs 15379, 23694, 25834, 27903, 27904, 9281 (housekeeping/rRNA in all four libraries) (Table S1). Four Llon_contigs could not be annotated (Llon_contigs 30589, 31202, 34793 and 4383) (Table S1 second sheet). There was no significant difference between SS1, SS2 and PP2 in the number of mapped-reads 5231 (Table S6). The number of mapped-reads 15379, 23694, 25834, 27903, 27904 and 9281 was significantly different in all four samples (Table S6). There was no significant difference between SS1, PP1 and PP2 in the number of mapped-reads 10948 (Table S6). The number of mapped-reads 32328 was significantly higher in SS2 with respect to the other three samples, whereas the number of mapped-reads 33875 was significantly higher in PP1 with respect to the other three samples (Table S6).

Eight Llon_contigs were identified exclusively in PP1, SS1 and SS2: Llon_contig 15118 (copper transmembrane transport in all three libraries; see Discussion), Llon_contig 21297 (stress, immunity and resistance only in SS1 and SS2; see Discussion), Llon_contig 32976 (stress, immunity and resistance in all three libraries; see Discussion), Llon_contig 27608 (cellular and metabolic processes only in SS2; see Discussion) and Llon_contigs 21934, 23004, 23764 and 5929 (which could not be annotated) (Table S1). The number of mapped-reads 15118 was significantly higher in PP1 with respect to both SS1 and SS2, and these reads were also significantly higher in SS1 with respect to SS2 (Table S7). There was no significant difference in the number of mapped-reads 32976 in all three samples (Table S7).

Two Llon_contigs which could not be annotated, 13814 and 28022, were identified exclusively in PP1, PP2 and SS2 (Table S1 second sheet).

The only Llon_contig identified exclusively in PP2, SS1 and SS2, was Llon_contig 32940 (cell motility and cytoskeletal structure only in SS1 and SS2; see Discussion) (Table S1). There was no significant difference in the number of mapped-reads 32940 between SS1 and SS2 (Table S7).

Nineteen Llon_contigs were identified exclusively in females (SS1 and PP1): Llon_contigs 10107, 11303, 11400, 12345, 12467, 12664, 13207, 13812, 14207, 15123, 16804. 18462, 22415, 28581, 4366, 4960, 5018, 5707 and 5826 (Table S1 second sheet). Of these, only 5 were partially annotated via *in silico* sequence analysis of the corresponding mapped-reads: Llon_contig 14207 (stress, immunity and resistance; see Discussion), Llon_contig 13812 (cell motility and cytoskeletal structure only in SS1; see Discussion), Llon_contig 10107 (others/salivary mucin; see Discussion), Llon_contig 5826 (cellular and metabolic processes) and Llon_contig 28581 (cellular and metabolic processes only in SS1; see Discussion) (Table S1). There was no significant difference between SS1 and PP1 in the number of mapped-reads 14207, 10107 and 5826 (Table S8).

Only two Llon_contigs were identified exclusively in males (SS2 and PP2): Llon_contigs 10010 (which could not be annotated) and 32330 (cell motility and cytoskeletal structure; see Discussion) (Table S1). There was no significant difference in the number of mapped-reads 32330 between samples (Table S8).

Fifteen Llon_contigs were identified exclusively in EVL males and females (SS2 and SS1), of which the following were partially annotated via *in silico* sequence analysis of the corresponding mapped-reads: Llon_contig 10109 (cellular and metabolic processes in SS1, and stress, immunity and resistance in SS2; see Discussion), Llon_contig 17984 (development), Llon_contig 34935 (cell motility and cytoskeletal structure; see Discussion) and Llon_contig 8847 (stress, immunity and resistance only in SS2; see Discussion) (Table S1). The rest of the Llon_contigs could not be annotated (Llon_contigs 1088, 19724, 20345, 23188, 25058, 25445, 29662, 3422, 5650, 638 and 7774) (Table S1 second sheet). There was no significant difference between SS1 and SS2 in the number of mapped-reads 17984 and 34935 (Table S8).

Sixteen Llon_contigs were identified exclusively in NEVL males and females (PP2 and PP1): Llon_contigs 15120, 30317, 30941, 11133, 9807, 4629, 34788, 33652, 28700, 28363, 27672, 27030, 25655, 21398, 16077 and 13642 (Table S1 second sheet). Seven of these were partially annotated: Llon_contig 15120 (copper transmembrane transport; see Discussion), Llon_contig 30317 (cellular transport), Llon_contig 34788 (cellular and metabolic processes only in PP1; see Discussion), Llon_contig 25655 (cellular and metabolic processes only in PP2; see Discussion), Llon_contig 33652 (cell motility and cytoskeletal structure; see Discussion) and Llon_contigs 21398 and 13642 (others/DNA integration; see Discussion) (Table S1). There was no significant difference between PP1 and PP2 in the number of mapped-reads 13642, 21398, 30317 and 33652, but mapped-reads 15120 were significantly overrepresented in NEVL females (Table S8).

Six Llon_contigs were identified exclusively in EVL females (SS1) and NEVL males (PP2): Llon_contigs 6260, 4238, 27738, 14835, 13641 and 10464 (Table S1 second sheet). Three of these Llon_contigs were partially annotated: Llon_contig 6260 (stress, immunity and resistance; see Discussion), Llon_contig 14835 (stress, immunity and resistance only in PP2; see Discussion) and Llon_contig 13641 (cellular and metabolic processes only in PP2; see Discussion) (Table S1). There was no significant difference between SS1 and PP2 in the number of mapped-reads 6260 (Table S8).

Seventeen Llon_contigs were identified exclusively in NEVL females (PP1) and EVL males (SS2): Llon_contigs 25465, 35303, 3400, 30802, 30603, 29416, 26564, 13578, 832, 6021, 35043, 34558, 33574, 2982, 29461, 28972 and 1068 (Table S1 second sheet). Of these, only Llon_contig 35303 was partially annotated (cellular and metabolic processes only in SS1; see Discussion) (Table S1).

## Discussion

This is the first study to perform a comprehensive unbiased comparative transcriptomic analysis of wild adult male and female *Lu. longipalpis*, integrating environmental conditions and taxa previously identified in these sand flies [22] in the final analysis. Total RNA extracted from wild adult male and female *Lu. longipalpis* from an endemic (Posadas, Misiones) and a non-endemic (Lapinha Cave, Minas Gerais) VL location in Argentina and Brazil, respectively, was submitted to sequence-independent amplification [25] and high-throughput pyrosequencing [32]. Due to the fact that total RNA was amplified using a modified sequence-independent amplification protocol [25] and, for this, first-strand reverse transcription was initiated with a random octamer linked to a specific primer sequence, an unbiased random collection of transcripts was obtained for each sample. Furthermore, transcripts from this study were initially blasted against a preliminary version of the *Lu. longipalpis* genome (downloaded from http://www.hgsc.bcm.tmc.edu) and, since the latter is as yet unannotated, subsequent BLAST and Blast2GO analyses of the reads that mapped to Llon_contigs, enabled the partial annotation of nearly a third of the Llon_contigs identified by sequence homology (Table S1 first sheet). Of these partially annotated Llon_contigs, nearly 50% returned no significant hits when the corresponding mapped-reads were blasted against the EST database (est-others), and almost 90% showed no significant hits when the corresponding mapped-reads were blasted against the non-redundant nucleotide sequence and 16S ribosomal RNA database (nt16S) (Table S1 first sheet). This confirmed that, with the chosen approach, not only were novel transcripts generated which, at the time this data was analysed had not been published in previous transcriptomic or genomic studies or been submitted to publicly available databases, but also that it was possible to assign putative functions to some of these novel transcripts.

Although previous studies have reported *Lu. longipalpis* ESTs for whole body [16], midgut [17], [18], salivary gland [19], [20] and, more recently, male reproductive organs [21], there is still need for more data, particularly from wild specimens in their natural environments. One of the goals of this work was to characterise the expression profile of wild adult male and female *Lu. longipalpis* from endemic and non-endemic VL locations. The unbiased transcriptomic analysis of these whole insects enabled the identification of transcripts putatively related to sand fly interactions with their natural environments and with previously associated taxa [22].

### Comparative analysis of the transcripts that mapped to Llon_contigs

Since amplification of total RNA in this study was random and not sequence specific, sequence homology of the collection of transcripts was also randomly variable (i.e. not associated with specific genetic regions) and, for those transcripts that showed homology to the same gene, only exceptionally did they show homology to the same region of that particular gene (data not shown). Furthermore, given the Llon_contigs have an average length of 7.5 Kb (Llon_contigs N50  =  7.5 Kb; http://www.hgsc.bcm.tmc.edu/collaborations/insects/sandfly/latest_assembly/README), transcripts mostly showed homology to different segments of the identified Llon_contigs. Due to this, in some cases it was possible to distinguish putative members of multigene families. For example, mapped-reads 32328 showed homology to different muscle actins (actins A2, C2, E2) and to beta-actin (Table S1). Moreover, mapped-reads 32974 showed homology to various members of the Vg family such as Vg precursor, Vg A1 and Vg C2, among others (Table S1). Conversely, in other cases transcripts which mapped to different Llon_contigs showed homology to the same gene, as was the case for Ctr1 (mapped-reads 15118 and 15120) and nesprin 1 (mapped-reads 35571 and 35572) (Table S1). In yet other cases, such as mapped-reads 15380 and 27608, some of the mapped-reads were associated with a putative biological function whereas others (that showed homology to the same Llon_contig) returned no significant hits (Table S1). In this context, some of the Llon_contigs which were identified in more than one sample, had different *in silico* annotation in the various libraries. These cases are mentioned below. Llon_contigs that are not indicated here showed consistent mapped-read homology search results (i.e. homology to the same gene) in all the libraries they were identified in (Table S1).

Of the 15 Llon_contigs that were identified in all four samples, mapped-reads 15380 only showed homology to a senescence-associated protein in PP2. In the rest of the samples the homology search returned no significant hits. Mapped-reads 10948 showed homology to a choline transporter-like protein in SS1, PP1 and PP2, but showed no significant hits in SS2. Mapped-reads 5231 showed homology to apolipophorin in SS1, SS2 and PP2, but the homology search returned no significant hits for PP1 (Table S1).

Of the eight Llon_contigs that were identified exclusively in PP1, SS1 and SS2, mapped-reads 21297 showed homology to cytochrome p450 in SS1, to a senescence-associated protein in SS2 and showed no significant hits in PP1. Mapped-reads 27608 showed homology to an SRP-dependent cotranslational protein in SS2 and returned no significant hits for SS1 and PP1 (Table S1).

Llon_contig 32940 was the only Llon_contig identified in PP2, SS1 and SS2. Mapped-reads 32940 showed homology to the myosin heavy chain in SS1 and SS2 and returned no significant hits for PP2 (Table S1).

Of the 19 Llon_contigs that were identified exclusively in females, mapped-reads 13812 showed homology to Sallimus gene, isoform L (muscle protein) in SS1 but returned no significant hits for PP1. Mapped-reads 28581 showed homology to purine biosynthesis protein 6 in SS1 and returned no significant hits for PP1 (Table S1).

Of the 15 Llon_contigs that were identified exclusively in EVL males and females (SS2 and SS1), mapped-reads 10109 showed homology to a protein involved in zinc ion binding in SS1 and to microtubule-associated serine/threonine-protein kinase 2 in SS2. Mapped-reads 8847 showed homology to a protein involved in chitin metabolic process in SS2 and returned no significant hits for SS1 (Table S1).

Of the 16 Llon_contigs that were identified exclusively in NEVL males and females (PP2 and PP1), mapped-reads 34788 showed homology to a neural stem cell-derived dendrite regulator in PP1 and returned no significant hits for PP2. Mapped-reads 25655 showed homology to transmembrane protein 65 in PP2 and returned no significant hits for PP1 (Table S1).

Of the 6 Llon_contigs that were identified exclusively in EVL females (SS1) and NEVL males (PP2), mapped-reads 14835 showed homology to a protein related to behavioural response to ethanol in PP2 and returned no significant hits for SS1. Mapped-reads 13641 showed homology to rabconnectin in PP2 and returned no significant hits for SS1 (Table S1).

Of the 17 Llon_contigs that were found exclusively in NEVL females (PP1) and EVL males (SS2), mapped-reads 35303 showed homology to an O-linked n-acetylglucosamine transferase in SS2 and returned no significant hits for PP1 (Table S1).

### Distinctive features of the expression profiles in EVL and NEVL samples

Transcripts (mapped-reads and read-nt16S) identified in EVL and NEVL samples were classified into categories according to their associated putative biological functions in each library (Figure 2). The most distinguishing characteristics of the different expression profiles are discussed here.

Transcripts specifically associated with environmental stress, immunity and resistance to xenobiotics were significantly overrepresented in EVL samples (SS1 and SS2) with respect to NEVL samples (PP1 and PP2) (Figure 2A). Most of the transcripts showed homology to inducible heat shock genes (HSP), reactive oxygen species (ROS)-scavenging enzymes, senescence associated proteins, autophagy-related proteins, mitogen-activated protein kinases (MAPKs), detoxification genes (cytochrome P450, arsenite transport), genes involved in resistance to xenobiotics (ATP-binding cassette (ABC) transporters, P450), serine protease inhibitors (serpins) and thioester-containing proteins (TEPs), among others (Tables S9–S12). The most significant transcripts are discussed in detail below.

Inducible HSPs are expressed at extremely low levels under normal conditions, but their transcription and translation increase rapidly in response to various stressors [33], [34], [35]. Heat shock protein 70 (HSP70) gene expression has been shown to increase in *Chironomus tentans* due to the effect of chemical (environmental pollutants including alkyl phenols, pesticides and heavy metals) and physical (heat shock and hypoxia) stressors [36]. HSP70 was identified in EVL males and females and in NEVL females. In females from both locations, mapped-reads 14207 showed homology to HSP70. Interestingly, in EVL males HSP70 was identified by sequence homology of a specific read-nt16S (not a mapped-read) and, thus, in that sample was not mapped to any Llon_contig (Tables S9, S10 and S11).

A mechanism of host defence against pathogens involves the deliberate production of various kinds of reactive oxygen species (ROS) [37], [38]. Blood-feeding insects also have to neutralise the actions of free heme which can generate ROS. Excessive release of ROS damages lipids, proteins and DNA [39], which leads to oxidative stress, loss of cell function and programmed cell death [40]. Blood-feeding insects possess a range of antioxidant enzyme systems including superoxide dismutase, catalase (which reduces H2O2 to H2O) and peroxidases of various kinds [41]. Previous studies in blood-feeding insect vectors have shown that ROS play an important role in fecundity [42], [43] and survival [37], [44], [45]. Biological damage related to ROS production has also been implicated in the process of ageing in dipterans. Previous studies on *Drosophila melanogaster* have shown that oxidative stress increases with age, while antioxidant enzyme activity decreases over time [46], [47], [48]. Furthermore, a recent study in *Lu. longipalpis* not only showed that fecundity and catalase expression decreased with age, but also incriminated catalase as an important component in the loss of fecundity [43]. Catalase was identified in EVL males and females and in NEVL females (Tables S9, S10 and S11). Interestingly, in all the samples, specific read-nt16S (i.e. not mapped-reads) showed homology to this gene, which was thus not mapped to any Llon_contig.

Cells entering a state of senescence undergo a permanent cell cycle arrest which is accompanied by a set of functional and morphological changes. Normal cells that are exposed to various physiologic stresses rapidly enter into a state of senescence, doing so within a period as short as several days [49]. Such stresses include DNA-damaging agents, oxidative stress and other metabolic perturbations [49], [50], [51], [52]. It is thought that various types of intrinsic and extrinsic stress stimuli can activate the senescence program and, whether this occurs rapidly or gradually following a period of proliferation, is determined mainly by the combined levels of those stresses [53]. Senescence-associated genes were identified in EVL and NEVL males, but these transcripts were mapped to different Llon_contigs: mapped-read 21297 in SS2 and mapped-read 15380 in PP2 (Tables S10 and S12). Granted senescence-associated genes encode diverse proteins, the fact that these transcripts mapped to more than one Llon_contig was not surprising.

Beclin is required for autophagy, embryogenesis and normal tissue homeostasis [54]. Autophagy is a complex catabolic program for lysosomal degradation of proteins and other subcellular constituents. It is often activated in response to nutrient deprivation, which leads to a recycling of organelles and other cytoplasmic substances to provide metabolic precursors. Failure to activate autophagy in response to nutrient deprivation, or its constitutive activation in response to stress, can lead to cell death. For this reason, autophagy is sometimes referred to as a second form of programmed cell death. Furthermore, autophagy and apoptosis are often activated together in response to stress [55], [56]. Beclin was only identified in SS1 by sequence homology of mapped-read 23288 (Table S9).

BRCA1 (BReast-CAncer susceptibility gene 1) and BRCA2 are tumor suppressor genes. Mutant phenotypes for these genes predispose to breast and ovarian cancers. BRCA proteins are involved in a multitude of pivotal cellular processes but, in particular, both genes contribute to DNA repair and transcriptional regulation in response to DNA damage. Recent studies suggest that BRCA proteins are required for maintenance of chromosomal stability, thereby protecting the genome from damage. New data also show that BRCAs transcriptionally regulate some genes involved in DNA repair, the cell cycle and apoptosis [57]. BRCA2 was identified in EVL females and NEVL males by sequence homology of mapped-reads 6260 (Tables S9 and S12).

Mismatch repair (MMR) systems play a central role in promoting genetic stability by repairing DNA replication errors, inhibiting recombination between non-identical DNA sequences and participating in responses to DNA damage [58]. MutS protein, a member of the ABC ATPase superfamily, recognises mispaired and unpaired bases in duplex DNA and initiates mismatch repair [59]. Transcripts that showed homology to the DNA mismatch repair MutS family and which mapped to Llon_contig 27951, were only found in PP1 (Table S11).

The peritrophic matrix (PM) forms a layer composed of chitin and glycoproteins that lines the insect midgut lumen [60], [61], which protects the midgut epithelium from abrasive food particles and microbes. Studies in insects have suggested that the PM plays a role in the defence against ingested pathogens [62]. Moreover, different genes involved in chitin metabolism have been found to affect parasite development [63], [64]. In this study, proteins involved in the chitin metabolic process were identified in EVL males by sequence homology of mapped-read 8847 (Table S10).

The mitogen-activated protein kinase (MAPK) cascade is an evolutionarily conserved signalling mechanism involved in processes as diverse as apoptosis, cell fate determination, immune function and stress response. An increase in the activity of two *Drosophila* MAPK genes (D-p38a and D-p38b) was observed in *Drosophila* cell lines in response to a variety of environmental stimuli including osmotic shock, heat shock, oxidative stress, immune stimulation, serum starvation and UV radiation [65], [66]. MAPKs were identified in SS2 and PP1 by sequence homology of mapped-reads 17958 and 7732, respectively (Tables S10 and S11). Due to their involvement in diverse processes, the fact that they mapped to different Llon_contigs was not surprising.

Rapid and specific intracellular protein degradation is instrumental in the control of cell cycle transitions, developmental programs and responses to environmental signals. Most proteins with very short half-lives are degraded by the ubiquitin proteolytic system, which catalyses the covalent attachment of polyubiquitin chains to substrate proteins [67]. Polyubiquitinated proteins are then captured rapidly by the 26S proteasome, an abundant, self-compartmentalised protease particle [68]. Specificity in the ubiquitin system is determined largely at the level of substrate recognition, a step that is mediated by E3 ubiquitin ligases. A new class of E3 ubiquitin ligases called SCF complexes are composed by different subunits (Skp1, Rbx1, Cdc53) and F-box proteins. The substrate specificity of SCF complexes is determined by the interchangeable F-box protein subunit [69]. Transcripts that were found in PP1, which mapped to Llon_contig 16604, were associated with the 26S proteasome regulatory subunit S3. Transcripts identified in SS1, which mapped to Llon_contigs 1520 and 7838, showed homology to an E3 ubiquitin ligase and an F-box only protein, respectively. Transcripts found in PP2, that mapped to Llon_contig 4021, showed homology to putative uncharacterised proteins containing F-box domains (Tables S9, S11 and S12).

Arsenic is toxic to all living organisms and the main sources which contribute to arsenic contamination in the environment include human activities such as the widespread application of arsenical insecticides, pesticides, herbicides, defoliants and wood preservatives [70], [71]. Arsenic has been defined as a group 1 carcinogen and is placed in the highest health hazard category [72], [73]. At the cellular level, arsenic toxicity depends to a large extent on the nature of the arsenical. In particular, as arsenite has a high binding affinity for the sulfhydryl groups found in cysteine, this disrupts protein structure and protein–protein interactions. Consequently, it affects many key metabolic processes such as fatty acid metabolism, glucose uptake and glutathione production [74]. In addition, the binding ability of arsenite to glutathione can lead to glutathione depletion and therefore increased levels of damaging ROS [75]. Transcripts that mapped to Llon_contig 33055, showed homology to arsenite transport proteins in EVL males (Table S10).

Serine proteases and their associated regulatory serine protease inhibitors, which belong to the serpin superfamily, play critical roles in the regulation of the invertebrate innate immune responses [76], [77], [78]. Transcripts that showed homology to serpins were found in EVL females and males (SS1 and SS2) and mapped to Llon_contigs 24258 and 30122, respectively (Tables S9 and S10).

The thioester-containing protein (TEP) family is represented in many metazoa. It encodes proteins that play an important role in immune responses as part of the complement system and as the universal protease inhibitors, alpha 2-macroglobulins. A hallmark of the family is the conserved thioester (TE) motif. After proteolytic activation, TEPs use TE for binding covalently to a nearby target, which is then cleared by phagocytic cells or destroyed by the membrane attack complex [79]. A member of this family, TEP4, was found to be up-regulated in *Plasmodium* infected mosquitoes [80]. Transcripts identified in EVL females (SS1), which mapped to Llon_contig 26544, showed homology to TEP3 (Table S9).

ABC transporters are involved in prokaryotic and eukaryotic development of resistance to multiple drugs. Moreover, an elevated expression of ABC transporters has been linked to insecticide resistance in several species [81], [82], [83], [84], [85], but the physiological mechanism by which these transporter proteins act to reduce insecticide susceptibility is unknown. Transcripts that showed homology to ABC transporters were identified in EVL males and females and NEVL females. These transcripts mapped to Llon_contigs 3834 and 8325 in SS1, to Llon_contig 17957 in SS2 and to Llon_contig 6659 in PP1 (Tables S9, S10 and S11). Due to their involvement in diverse processes, the fact that they mapped to various Llon_contigs was not unexpected.

Many P450 proteins are specialised in the metabolism of endogenous substrates (steroid hormones, lipids, etc.), but much of their notoriety has been associated with the detoxification of xenobiotics (natural products, drugs, pesticides, etc.). In insects, they have been involved in many cases of insecticide resistance [86], [87], [88] and, in particular, pyrethroid resistance [85], [89]. A transcript that mapped to Llon_contig 21297 and which showed homology to P450 was identified in SS1 (Table S9).

Posadas, the densely populated capital city of the province of Misiones, is located in the subtropical fields and grasslands ecoregion. In the Posadas area, this ecoregion contacts the Paranaense forest and has a savannah-type landscape. The trap was installed in the peridomicile of a worst-case scenario homestead (family homestead situated in a densely populated urban area with a spring of water, dense vegetation and abundant organic matter produced by domestic animals and humans) (Figure 2) [22]. Moreover, extensive peridomestic pyrethroid spraying, which has not yet shown a long-term effect on vector abundance, is performed by local officials in Posadas [90], and high levels of environmental pollutants including arsenic have also been reported. The fact that transcripts associated with environmental stress, immunity, detoxification and resistance to xenobiotics were significantly overrepresented in EVL samples could be related to a higher level of environmental stressors and, consequently, selective pressure, in Posadas city respect to Lapinha Cave (Figure 2). In particular, the identification of transcripts associated with ABC transporters and cytochrome p450, coupled with the widespread peridomestic spraying of pyrethroids, could be reflecting that the Posadas *Lu. longipalpis* population is developing resistance to this insecticide. In this sense, insecticide resistance in phlebotomine sand flies to date has been recorded in *P. papatasi* and *P. argentipes*, probably as a result of exposure to DDT used in anti-malarial spraying campaigns [91], [92], [93]. Moreover, a significant reduction in susceptibility to pyrethroids was recently reported for wild populations of *Lu. longipalpis* [94]. On the other hand, protist parasite sequences were previously found in these same samples, of which the vast majority were found in males (Figure 2) [22]. Nearly 90% of the identified apicomplexans corresponded to coccidians and the rest to gregarines (genera Ascogregarina and Psychodiella, 7%) and haemosporidians [22]. Since gregarines can reduce longevity and egg production in *Lu. longipalpis*, and the level of parasitaemia can reach over 80% in laboratory colonies [95], the overrepresentation of immunity and stress related transcripts in EVL samples was also likely related to the presence of these protist parasite sequences (Figure 2).

As mentioned previously, transcripts associated with stress, immunity and resistance were significantly underrepresented in NEVL males and females with respect to EVL samples (Figure 2). This result was in accordance with their environment of origin and with taxa previously associated with these sand flies [22]. Specifically, the Lapinha Cave is a network of interconnected caves located in the Sumidouro State Park. This is a protected area situated in a vast tropical savannah ecoregion called *cerrado* which is characterised by great plant and animal biodiversity (Figure 2) [22]. As it is a protected area, there is no record of insecticide spraying or environmental pollution. Furthermore, taxa previously associated with these sand flies included bacteria, fungi, plants and metazoa which were indicative of their feeding habits and behavioural patterns (Figure 2) [22]. Nevertheless, transcripts associated with stress, immunity and resistance were significantly overrepresented in NEVL females with respect to NEVL males (Figure 2). Since NEVL females were probably undergoing oogenesis after blood feeding (see below), this could account for the significant overrepresentation of these transcripts in PP1. Moreover, transcripts associated with mismatch repair functions (MutS protein) were exclusively identified in PP1 (Table S11), which could be related to an increased DNA replication activity. Additionally, various transcripts related to the cell cycle (cyclin, which was significantly overrepresented in PP1 (Table S6), and mitotic checkpoint protein MAD1) and others associated with regulation of transcription, translation, oogenesis and protein deacetylation, were also identified in NEVL females (Table S11), further supporting this possible scenario. In this context, catalase expression in NEVL females (Table S11) was likely more related to the bloodmeal intake than to environmental stress factors.

Actin, a ubiquitous and highly conserved protein that in many organisms is encoded by multigene families [96], was the only protein associated with cell motility and cytoskeletal structure that was identified in all samples. Diversity in actin gene structure and expression has been studied extensively in *D. melanogaster* [97]. The *D. melanogaster* genome contains six actin genes [98]: two are cytoplasmic and the other four are predominantly muscle isoforms [99], [100]. Mapped-reads 32328, which were identified in all four samples, showed homology to actin muscle isoforms (actins E2, C2 and A2) and beta-actin (Table S1). Mapped-reads 32330, identified in EVL and NEVL males (SS2 and PP2), showed homology to cytoplasmic actin (Table S1). Other transcripts related to cell motility and cytoskeletal structure included mapped-reads 24125, 32134, 32354, 32940, 33652, which showed homology to myosin; mapped-read 34935 showed homology to titin, a giant filamentous protein which in vertebrates spans the distance between the Z- and M-lines of the muscle sarcomere [101]; mapped-reads 11598 and 11599 showed homology to dynein; mapped-read 24492 showed homology to pinin, a protein that in mammals has been associated with the desmosome-intermediate filament complex [102]; and mapped-reads 35571 and 35572 showed homology to nesprin, which belongs to a novel family of nuclear and cytoskeletal proteins that are highly versatile tissue specific intracellular protein scaffolds [103] (Table S1). These transcripts were significantly overrepresented in EVL samples with respect to NEVL samples (Figure 2).

Transcripts associated with lipid transport were significantly overrepresented in NEVL females (Figure 2) and vitellogenin (Vg), which was found exclusively in PP1, was the most distinctive protein in this category. Vg is a large phospholipoglycoprotein encoded by a small family of nearly-identical genes. Insect Vg harbours potential sites for lipidation, glycosylation and phosphorylation and is internalised by developing oocytes where it is proteolytically cleaved to generate vitellin, a nutrient source for the developing embryo [104], [105]. The blood meal provides amino acids and lipids that are transferred through midgut cells to the hemolymph and signal to initiate massive synthesis of nutrient transport proteins in the mosquito fat body [106]. These lipid transport proteins include lipophorin and Vg. Both proteins are secreted into the hemolymph and transported to the ovaries. In this context, since a chicken was kept in the Lapinha Cave (capture site of NEVL males and females) to attract the sand flies and as a source of food, it is very probable the females had taken a blood meal, because *Gallus gallus* sequences were previously identified in these same sand flies [22]. Given vitellogenesis is normally concomitant with the digestion of a blood meal [107], this possible scenario would account for the overrepresentation of Vg transcripts in NEVL females (Table S11).

Copper transmembrane transport transcripts, namely mapped-reads 15118 and 15120, showed homology to Ctr1 (high-affinity copper uptake protein 1) and were significantly overrepresented in NEVL females (Figure 2). Since copper is the cofactor of a variety of proteins, it is required for a number of biological reactions including respiration (cytochrome c oxidase), free radical defense (superoxide dismutase), maturation of connective tissues (lysyl oxidase), neurotransmitter biosynthesis (dopamine B-monooxygenase) and iron homeostasis (ceruloplasmin) [108], [109]. On the other hand, its ability to generate hydroxyl radicals, which can be incorporated into proteins inappropriately and interfere with the homeostasis of other metals, can induce toxic effects in living organisms [110], [111]. A previous study showed that lizard oocytes accumulate both copper and zinc during the preparatory phases of oogenesis and, although the physiological role of copper in oocytes remained unknown, there was evidence for its importance in embryonic and fetal development. This study also revealed that the transcript encoding Ctr1 increased during oocyte growth and accumulated mostly in eggs, suggesting that Ctr1 in eggs can be part of the copper trafficking machinery responsible for uptake and storage of copper that is used during embryonic development [112]. These results would correlate with the overrepresentation of Ctr1 transcripts in NEVL females (Table S11 and Figure 2), since they were likely undergoing oogenesis at the time of sampling.

Transcripts associated with cellular and metabolic processes showed distinct patterns in every sample. As mentioned previously, various transcripts associated with oogenesis, transcription regulation, translation and protein deacetylation were identified in PP1. This was in accordance with the probable ongoing process of vitellogenesis in NEVL females at the time of sampling, inferred from the overrepresentation of Vg and Ctr1 transcripts (Table S11) and the identification of an elevated number of chicken sequences in these females [22].

Transcripts that showed homology to cytochrome c oxidase subunit I (CCOI) were significantly overrepresented in NEVL males (Table S12) with respect to the rest of the samples. Interestingly, CCOI was identified by sequence homology of read-nt16S (not mapped-reads) in all the samples and, thus, was not mapped to any Llon_contig (Tables S9–S12). Cytochrome c oxidase (CCO) is a large transmembrane protein complex found in the mitochondrion. As the last key enzyme in the respiratory electron transport chain of mitochondria, CCO plays an important role in ATP generation by oxidative phosphorylation. In eukaryotes, three subunits (I–III) of CCO are encoded by mitochondrial DNA, forming the functional catalytic core of the enzyme [113]. Overexpression of the CCOI gene has been linked to pyrethroid resistance in the German cockroach [114] and to praziquantel resistance in *Schistosoma mansoni*, a drug used to control schistosomiasis [115]. Overexpression of CCOII has been involved in the development of resistance to methotrexate, a widely used drug in cancer chemotherapy, in Chinese hamster ovary cells [116]. More recently, permethrin was found to specifically induce overexpression of CCOIII in *A. aegypti* [117], [118]. Furthermore, inhibition of CCO by mitochondrial antisense RNA induced cell death in human cell lines [119]. In this context, the overrepresentation of CCOI in PP2 was surprising because there is no evidence suggesting that the Lapinha sand fly population has been significantly exposed to insecticides [94] and, to our knowledge, no insecticides were being applied in the Lapinha Cave area at the time of sampling. Furthermore, as was mentioned previously, the Lapinha Cave is situated in the Sumidouro State Park and is a protected area with no evidence of insecticide applications. Moreover, no other transcripts associated with insecticide resistance were identified in these samples and very few were found in NEVL females. Nonetheless, since these are environmental samples and notwithstanding most variables were accounted for, the application of insecticides cannot be dismissed.

Various transcripts related to purine metabolism were identified in EVL females, together with other transcripts associated with carbohydrate and glycogen metabolic processes, glycogen biosynthetic process, protein modification processes, transcription and translation (Table S9). Since we previously identified human sequences in this sample [22], it is plausible that these female sand flies had also taken a bloodmeal. In this context, as purines and proteins are metabolised into urate during the digestion of the bloodmeal [126], this could account for the higher proportion of transcripts associated with purine metabolism.

In EVL males, several of the transcripts in this category were associated with calcium ion binding. This was likely related to the significantly high number of transcripts associated with cell motility and cytoskeletal structure in this sample (actin, titin, myosin, among others; see Table S10).

Putative salivary proteins were identified in females from both locations. Mapped-reads 10107 showed homology to a putative salivary mucin of unknown function in EVL and NEVL females (Tables S9 and S11). This mucin belongs to a family that could encode glycoproteins, act as lubricant of salivary canals and be involved in the interaction with and invasion of mammalian host cells [120]. On the other hand, mapped-read 5162 showed homology to a putative yellow related-protein in NEVL females (Table S11). The yellow protein family is one of the most abundant proteins found in sand fly saliva, even though its function in the saliva and importance for blood feeding are unknown [20]. In *Drosophila* it has been related to pigmentation and male sexual behaviour and, in *Ae. Aegypti*, to the activity of a dopachrome converting enzyme from the melanisation pathway [121]. Melanisation plays important physiological roles in insects including cuticle and egg chorion hardening, wound healing and immune responses against parasites in insects [121]. Infected sand flies inject saliva together with the parasite into the mammalian host during the bloodmeal intake. This saliva not only facilitates blood feeding [122] but also affects the establishment of the parasite within the vertebrate host. Small quantities of *Lu. longipalpis* saliva have been shown to exacerbate *Le. major* infection in mice [123], [124]. Conversely, immune responses to sand fly saliva have been shown to protect against *Leishmania* infection [125], and antibodies to maxadilan, a salivary protein from *Lu. longipalpis*, protected mice against *Le. major* infection [126]. A recent comparative salivary gland transcriptomic analysis of VL vectors [20] identified families of salivary proteins common among all the sand flies studied, proteins that were genus specific and putative species specific proteins. These results suggested that genus- or species-specific salivary proteins could be necessary for the development of a vector-based vaccine. In this context, the identification of transcripts associated with putative salivary proteins in wild populations of *Lu. longipalpis* resulted of interest.

In SS1, mapped-read 24422 showed homology to a chorion peroxidase (Table S9). This enzyme is upregulated during the last stage of oogenesis by the presence of endogenously synthesised and released hydrogen peroxide molecules. Following protein-protein interactions and intercalation events during the chorion formation process, this enzyme hardens the chorion to produce a functional and completely assembled chorion [127]. Furthermore, there is strong evidence suggesting that the mechanism of chorion hardening is highly conserved among all Diptera [127], [128]. Thus, chorion peroxidase is a putative protein to be targeted *in vivo* for the development of alternative biocontrol protocols.

In insects, neuropeptides not only regulate the functioning of endocrine glands, but also a wide range of physiological and developmental processes [129]. Neuropeptides and peptide hormones are often generated from larger precursor proteins via a complex series of posttranslational modifications. In EVL females, mapped-read 34912 showed homology to prohormone convertase 2 (PC2) (Table S9), an enzyme involved in the proteolytic maturation of neuropeptide precursors into mature peptides that act as neurotransmitters, neuromodulators or neurohormones [130].

Novel insecticide targets potentially exist among the arthropod G protein-coupled receptors (GPCRs). These proteins comprise a large family of membrane-bound molecules that mediate critical biological processes such as neurotransmission, vision and hormonal regulation, among others [131], [132]. GPCRs are extensively targeted for drug development in humans (around 40% of prescription pharmaceuticals interact with these receptors) [133], [134]. Furthermore, more than 100 different GPCRs have been identified in the genomes of many insect species [135], [136] and these studies have provided a basis for the functional characterisation of GPCRs and their prioritisation as potential subjects for insecticide development [137]. A GPCR was identified in PP1 by sequence homology of a specific read-nt16S and was thus not mapped to any Llon_contig (Table S11).

Transposable elements are DNA sequences that have the ability to replicate within a genome using a variety of mechanisms [138]. They are present in almost all eukaryotic genomes and play an important role in genome evolution by creating genetic variation through their mobility [139], [140]. Transposable elements can be divided into two classes based on their replication mechanism: retrotransposons (class I) and DNA transposons (class II) [138]. While retrotransposons use an RNA intermediate for transposition, DNA transposons use a DNA intermediate. Because of their replication mechanism, retrotransposons are generally present in larger numbers than DNA transposons and can reach very high copy numbers. They also show a broader phylogenetic distribution [141]. Retrotransposons were identified in NEVL males and females by sequence homology of mapped-reads 13642 and 21398 in both samples and of mapped-read 1948 in NEVL females (Tables S11 and S12). Their presence in both NEVL samples could be related to an ongoing process of genomic variation in that *Lu. longipalpis* population at the time of sampling.

In order to better appraise the results presented in this study, some of the limitations inherent to the chosen approach must be mentioned. An obvious point is the high proportion of transcripts with homology to insect rDNA (mainly *Lu. longipalpis*) (∼86%) which undoubtedly masked the expression of other genes. Nevertheless, the purpose of this analysis was to concomitantly integrate wild-caught sand fly transcriptome data with the information of taxa that we previously identified by sequence homology in these samples [22] and environmental conditions, to obtain a novel and unique insight into wild adult (male and female) *Lu. longipalpis* gene expression profiles under natural conditions. In this sense, if mRNA only had been extracted and amplified (i.e. if an exhaustive transcriptomic analysis had been performed), this would have precluded the concomitant identification of an important fraction of the associated taxa [22]. Another point which has been mentioned elsewhere [22], is that homology searches are circumscribed to the number and quality of sequences in the databases at the time of analysis. The relatively high number of sequences which showed no significant hits after the first and second stages of analysis (114,664 reads, 12.46%) (Figure 1) was a clear indication of this. Moreover, if the query corresponds to a given organism or gene that has not yet been sequenced, the hit will probably coincide with a closely related organism or gene found in the database. Notwithstanding and given this situation, the results from the homology search will provide a close approximation to the real case-scenario. Summarising and notwithstanding the aforementioned limitations, the chosen approach was appropriate to obtain a comprehensive view of the sand flies analysed here as environmental samples, ensuring no data was lost and enabling the identification of genes that could putatively influence sand fly development and which will become the target of ongoing studies to determine their significance in *Lu. longipalpis*.

The fact that nearly 50% of the partially annotated Llon_contigs returned no significant hits when the corresponding mapped-reads were blasted against the EST-others database, reveals that the chosen approach was effective for identifying novel transcripts. Moreover, the partial *in silico* annotation of various of the Llon_contigs via subsequent BLAST and Blast2Go analyses of the corresponding mapped-reads and, in particular, the mapping of specific Llon_contig regions to particular functions, further increases current knowledge of sand fly biology. Moreover, as the samples used in this study were randomly caught wild adult male and female specimens from EVL and NEVL locations, and the chosen strategy enabled the concomitant comparison of sand fly gene expression data with previously associated taxa and the environmental conditions, this study provides an invaluable description of sand fly genes that are being expressed in their natural environments under diverse conditions. This contributes a unique novel insight into sand fly biology under different environmental conditions, i.e. an endemic VL location (Posadas) with a high level of environmental stressors such as the widespread use of pyrethroids, contamination and parasite infection, and a non-endemic VL location (Lapinha Cave) that posed no obvious environmental stresses for the sand flies.

Sand fly control in Latin America relies mostly on pyrethroid insecticide spraying and impregnated bed nets [142]. However, because of an increasing trend in insecticide resistance induced by selection pressure, chemically based control of sand fly populations could become increasingly difficult [91], [92], [93], [94]. Other negative impacts of insecticide usage include accumulation of the insecticide within the environment and the widespread killing of non-target organisms. Thus, insecticides can create a long-term burden on species diversity and ecosystem sustainability [143]. In this context, double-stranded RNA (dsRNA) is an attractive alternative as a potential bioinsecticide because it avoids the negative effects of chemical insecticides. Specifically, it poses no risk of accumulation within the environment because it is readily degraded by ubiquitous bacterial enzymes. In addition, it is highly sequence-specific and can be designed to avoid nontarget species toxicity [143]. Within cells, dsRNA induces RNA interference (RNAi), a naturally occurring process by which mRNA is silenced prior to translation by a large protein complex guided by complementary small interfering RNAs (siRNAs) that are generated from the original dsRNA molecules that induced the response [144]. The RNAi response can be induced experimentally through the delivery of dsRNA through a variety of means, including injection, topical application or oral delivery. For controlling insects in the field only remote methods of introduction can be utilised, such as topical application or oral delivery [145]. Recently, knockdown of a gene target through oral delivery of dsRNA was reported in adult *A. aegypti* [143]. Another study in this mosquito demonstrated that adult mortality could be induced by the topical application of dsRNA against IAP (inhibitor of apoptosis) [146]. Furthermore, dsRNA targeting chitin synthase mRNA was fed to *An. gambiae* larvae, resulting in gene silencing and significant mortality [147]. A recent study in *P. papatasi* showed that the injection of dsRNA to induce knockdown of PpChit1 (a midgut-specific chitinase presumably involved in the maturation/degradation of the PM in the gut of the sand fly after a bloodmeal) led to a significant reduction of *Le. major* within the gut [64].

To develop dsRNA as a means of sand fly population control, either alone or in conjunction with other control measures, gene targets that can induce physiological changes that affect survivorship, fecundity and/or behaviour must be identified. In this study, various such putative targets were identified in EVL and NEVL *Lu. longipalpis* specimens, which included transcripts associated to chitin metabolism, catalase, chorion peroxidase and GPCR, among others. Future studies will test the potential of these gene targets, with a special emphasis on avoiding off-target effects such as non-specific lethality or non-specific gene silencing, in order to maximise the effect of specific gene silencing for sand fly control.

## Supporting Information

Table S1 **Total Llon_contigs.** First sheet (Llon_contigs with annotation): This table shows the total number of Llon_contigs that were partially annotated by sequence analysis of their corresponding mapped-reads, identified in all the samples. The information provided for each Llon_contig includes: Llon_contigs that mapped-reads showed homology to; function category; BLASTX results (DB:uniprotKB and DB:nr); GO annotation, including biological process, molecular function and cellular component; BLASTN results against DB:est-others; and BLASTN results against DB:nt16S. At the bottom of the table the following information is provided: total number of annotated Llon_contigs considering all four samples; total number of annotated Llon_contigs that returned no significant hits when the corresponding mapped-reads were blasted against DB:est-others; and total number of annotated Llon_contigs that returned no significant hits when the corresponding mapped-reads were blasted against DB:nt16S. Second sheet (Total Llon_contigs comparison): This table shows the total number of Llon_contigs identified in all the samples, with or without partial *in silico* annotation. The information provided for each Llon_contig includes: mapped-read indicating the sample it was identified in; function category when applicable; a brief description of the hit against DB:uniprotKB, when applicable; and GO annotation when applicable, including biological process, molecular function and cellular component. Mapped-reads found in more than one sample are highlighted in bold. ST: Stress, immunity and resistance; ME: Cellular and metabolic processes; CY: Cell cycle and genome organisation; DE: Development; MO: Cell motility and cytoskeletal structure; TR: Cellular transport; LP: Lipid transport; Cu: Copper transmembrane transport; OT: Other; SS1: adult EVL females; SS2: adult EVL males; PP1: adult NEVL females; PP2: adult NEVL males.
(XLS)

Table S2 **SS1_Llon_contigs.** First sheet (SS1 annotated Llon_contigs): This table lists the Llon_contigs identified in SS1 (EVL females) that were partially annotated by in silico sequence analysis of the corresponding mapped-reads. The information provided for each Llon_contig includes: Llon_contig number; description of the best BLASTX hit against DB:uniprotKB; and GO annotation including biological process, molecular function and cellular component. BLASTN hits against DB:nt16S are only shown for those Llon_contigs whose corresponding mapped-reads overall showed homology to insect rDNA. Second sheet (Total SS1 Llon_contigs): This table lists the totality of the Llon_contigs identified in SS1 (EVL females), with or without partial in silico annotation. The information provided for each Llon_contig includes: Llon_contig number; description of the best BLASTX hit against DB:uniprotKB, when applicable; and GO annotation including biological process, molecular function and cellular component, when applicable. BLASTN hits against DB:nt16S are only shown for those Llon_contigs whose corresponding mapped-reads overall showed homology to insect rDNA.
(XLS)

Table S3 **SS2_Llon_contigs.** First sheet (SS2 annotated Llon_contigs): This table lists the Llon_contigs identified in SS2 (EVL males) that were partially annotated by in silico sequence analysis of the corresponding mapped-reads. The information provided for each Llon_contig includes: Llon_contig number; description of the best BLASTX hit against DB:uniprotKB; and GO annotation including biological process, molecular function and cellular component. BLASTN hits against DB:nt16S are only shown for those Llon_contigs whose corresponding mapped-reads overall showed homology to insect rDNA. Second sheet (Total SS2 Llon_contigs): This table lists the totality of the Llon_contigs identified in SS2 (EVL males), with or without partial in silico annotation. The information provided for each Llon_contig includes: Llon_contig number; description of the best BLASTX hit against DB:uniprotKB, when applicable; and GO annotation including biological process, molecular function and cellular component, when applicable. BLASTN hits against DB:nt16S are only shown for those Llon_contigs whose corresponding mapped-reads overall showed homology to insect rDNA.
(XLS)

Table S4 **PP1_Llon_contigs.** First sheet (PP1 annotated Llon_contigs): This table lists the Llon_contigs identified in PP1 (NEVL females) that were partially annotated by in silico sequence analysis of the corresponding mapped-reads. The information provided for each Llon_contig includes: Llon_contig number; description of the best BLASTX hit against DB:uniprotKB; and GO annotation including biological process, molecular function and cellular component. BLASTN hits against DB:nt16S are only shown for those Llon_contigs whose corresponding mapped-reads overall showed homology to insect rDNA. Second sheet (Total PP1 Llon_contigs): This table lists the totality of the Llon_contigs identified in PP1 (NEVL females), with or without partial in silico annotation. The information provided for each Llon_contig includes: Llon_contig number; description of the best BLASTX hit against DB:uniprotKB, when applicable; and GO annotation including biological process, molecular function and cellular component, when applicable. BLASTN hits against DB:nt16S are only shown for those Llon_contigs whose corresponding mapped-reads overall showed homology to insect rDNA.
(XLS)

Table S5 **PP2_Llon_contigs.** First sheet (PP2 annotated Llon_contigs): This table lists the Llon_contigs identified in PP2 (NEVL males) that were partially annotated by in silico sequence analysis of the corresponding mapped-reads. The information provided for each Llon_contig includes: Llon_contig number; description of the best BLASTX hit against DB:uniprotKB; and GO annotation including biological process, molecular function and cellular component. BLASTN hits against DB:nt16S are only shown for those Llon_contigs whose corresponding mapped-reads overall showed homology to insect rDNA. Second sheet (Total PP2 Llon_contigs): This table lists the totality of the Llon_contigs identified in PP2 (NEVL males), with or without partial in silico annotation. The information provided for each Llon_contig includes: Llon_contig number; description of the best BLASTX hit against DB:uniprotKB, when applicable; and GO annotation including biological process, molecular function and cellular component, when applicable. BLASTN hits against DB:nt16S are only shown for those Llon_contigs whose corresponding mapped-reads overall showed homology to insect rDNA.
(XLS)

Table S6 **FET Llon_contigs in 4 samples.** This table shows the statistical analysis between samples of the number of mapped-reads that were identified in all four samples (Fisher's Exact Test; p<0.05), for those samples in which the Llon_contigs showed consistent mapped-read homology search results (i.e. homology to the same gene or to members of the same multigene family). The table shows the Llon_contig reads mapped to; the number of reads that mapped to each Llon_contig for each sample; the library the mapped-reads were identified in; the statistical analysis between samples for those Llon_contigs that showed consistent mapped-read homology search results; and a brief description of the in silico annotation for those Llon_contigs. NA: not applicable; *: significant difference; ns: no significant difference; SS1: adult EVL females; SS2: adult EVL males; PP1: adult NEVL females; PP2: adult NEVL males.
(XLS)

Table S7 **FET Llon_contigs in 3 samples.** This table shows the statistical analysis between samples of the number of mapped-reads that were identified in three samples (Fisher's Exact Test; p<0.05), for those samples in which the Llon_contigs showed consistent mapped-read homology search results (i.e. homology to the same gene): PP1, SS1 and SS2; PP1, PP2 and SS2; PP2, SS1 and SS2. The table shows the Llon_contig reads mapped to; the number of reads that mapped to each Llon_contig for each sample; the library the mapped-reads were identified in; the statistical analysis between samples for those Llon_contigs that showed consistent mapped-read homology search results; and a brief description of the in silico annotation for those Llon_contigs. NA: not applicable; *: significant difference; ns: no significant difference; SS1: adult EVL females; SS2: adult EVL males; PP1: adult NEVL females; PP2: adult NEVL males.
(XLS)

Table S8 **FET Llon_contigs in 2 samples.** This table shows the statistical analysis between samples of the number of mapped-reads that were identified in two samples (Fisher's Exact Test; p<0.05), for those samples in which the Llon_contigs showed consistent mapped-read homology search results (i.e. homology to the same gene): PP1 and SS1; PP2 and SS2; SS1 and SS2; PP1 and PP2; SS1 and PP2; PP1 and SS2. The table shows the Llon_contig reads mapped to; the library the mapped-reads were identified in; the statistical analysis between samples for those Llon_contigs that showed consistent mapped-read homology search results; and a brief description of the in silico annotation for those Llon_contigs. NA: not applicable; *: significant difference; SS1: adult EVL females; SS2: adult EVL males; PP1: adult NEVL females; PP2: adult NEVL males.
(XLS)

Table S9 **SS1 transcript categories.** First Sheet (SS1 transcript categories): This table shows the total number of mapped-reads and read-nt16S in SS1 (EVL females) that were associated with a putative biological function, grouped according to the function category they were assigned to. The provided information includes: function category; transcript identification (mapped-read or read-nt16S); unique read name (name assigned to each read when the samples were pyrosequenced); read length; description of the best Blastx hit against DB:uniprotKB and its score and E-value; and GO annotation including biological process, molecular function and cellular component, when applicable. Second sheet (UniprotKB+GO+BLASTN): This table also shows the total number of mapped-reads and read-nt16S in SS1 (EVL females) that were associated with a putative biological function but, apart from including the UniprotKB hit for each transcript, also shows the EST-others BLASTN result for all these transcripts, the nt16S BLASTN result only for the selected read-nt16S and, additionally, transcripts are ordered alphabetically. The provided information includes: transcript identification (mapped-read or read-nt16S); unique read name (name assigned to each read when the samples were pyrosequenced); read length; description of the best Blastx hit against DB:uniprotKB and its score and E-value; GO annotation including biological process, molecular function and cellular component, when applicable; Blastn result against DB:est-others and its score and E-value, when applicable; Blastn result against DB:nt16S for each selected read-nt16S and its score and E-value, when applicable. ST: Stress, immunity and resistance; ME: Cellular and metabolic processes; CY: Cell cycle and genome organisation; DE: Development; MO: Cell motility and cytoskeletal structure; TR: Cellular transport; LP: Lipid transport; Cu: Copper transmembrane transport; OT: Other; SS1: adult EVL females.
(XLS)

Table S10 **SS2 transcript categories.** First Sheet (SS2 transcript categories): This table shows the total number of mapped-reads and read-nt16S in SS2 (EVL males) that were associated with a putative biological function, grouped according to the function category they were assigned to. The provided information includes: function category; transcript identification (mapped-read or read-nt16S); unique read name (name assigned to each read when the samples were pyrosequenced); read length; description of the best Blastx hit against DB:uniprotKB and its score and E-value; and GO annotation including biological process, molecular function and cellular component, when applicable. Second sheet (UniprotKB+GO+BLASTN): This table also shows the total number of mapped-reads and read-nt16S in SS2 (EVL males) that were associated with a putative biological function but, apart from including the UniprotKB hit for each transcript, also shows the EST-others BLASTN result for all these transcripts, the nt16S BLASTN result only for the selected read-nt16S and, additionally, transcripts are ordered alphabetically. The provided information includes: transcript identification (mapped-read or read-nt16S); unique read name (name assigned to each read when the samples were pyrosequenced); read length; description of the best Blastx hit against DB:uniprotKB and its score and E-value; GO annotation including biological process, molecular function and cellular component, when applicable; Blastn result against DB:est-others and its score and E-value, when applicable; Blastn result against DB:nt16S for each selected read-nt16S and its score and E-value, when applicable. ST: Stress, immunity and resistance; ME: Cellular and metabolic processes; CY: Cell cycle and genome organisation; DE: Development; MO: Cell motility and cytoskeletal structure; TR: Cellular transport; LP: Lipid transport; Cu: Copper transmembrane transport; OT: Other; SS2: adult EVL males.
(XLS)

Table S11 **PP1 transcript categories.** First Sheet (PP1 transcript categories): This table shows the total number of mapped-reads and read-nt16S in PP1 (NEVL females) that were associated with a putative biological function, grouped according to the function category they were assigned to. The provided information includes: function category; transcript identification (mapped-read or read-nt16S); unique read name (name assigned to each read when the samples were pyrosequenced); read length; description of the best Blastx hit against DB:uniprotKB and its score and E-value; and GO annotation including biological process, molecular function and cellular component, when applicable. Second sheet (UniprotKB+GO+BLASTN): This table also shows the total number of mapped-reads and read-nt16S in PP1 (NEVL females) that were associated with a putative biological function but, apart from including the UniprotKB hit for each transcript, also shows the EST-others BLASTN result for all these transcripts, the nt16S BLASTN result only for the selected read-nt16S and, additionally, transcripts are ordered alphabetically. The provided information includes: transcript identification (mapped-read or read-nt16S); unique read name (name assigned to each read when the samples were pyrosequenced); read length; description of the best Blastx hit against DB:uniprotKB and its score and E-value; GO annotation including biological process, molecular function and cellular component, when applicable; Blastn result against DB:est-others and its score and E-value, when applicable; Blastn result against DB:nt16S for each selected read-nt16S and its score and E-value, when applicable. ST: Stress, immunity and resistance; ME: Cellular and metabolic processes; CY: Cell cycle and genome organisation; DE: Development; MO: Cell motility and cytoskeletal structure; TR: Cellular transport; LP: Lipid transport; Cu: Copper transmembrane transport; OT: Other; PP1: adult NEVL females.
(XLS)

Table S12 **PP2 transcript categories.** First Sheet (PP2 transcript categories): This table shows the total number of mapped-reads and read-nt16S in PP2 (NEVL males) that were associated with a putative biological function, grouped according to the function category they were assigned to. The provided information includes: function category; transcript identification (mapped-read or read-nt16S); unique read name (name assigned to each read when the samples were pyrosequenced); read length; description of the best Blastx hit against DB:uniprotKB and its score and E-value; and GO annotation including biological process, molecular function and cellular component, when applicable. Second sheet (UniprotKB+GO+BLASTN): This table also shows the total number of mapped-reads and read-nt16S in PP2 (NEVL males) that were associated with a putative biological function but, apart from including the UniprotKB hit for each transcript, also shows the EST-others BLASTN result for all these transcripts, the nt16S BLASTN result only for the selected read-nt16S and, additionally, transcripts are ordered alphabetically. The provided information includes: transcript identification (mapped-read or read-nt16S); unique read name (name assigned to each read when the samples were pyrosequenced); read length; description of the best Blastx hit against DB:uniprotKB and its score and E-value; GO annotation including biological process, molecular function and cellular component, when applicable; Blastn result against DB:est-others and its score and E-value, when applicable; Blastn result against DB:nt16S for each selected read-nt16S and its score and E-value, when applicable. ST: Stress, immunity and resistance; ME: Cellular and metabolic processes; CY: Cell cycle and genome organisation; DE: Development; MO: Cell motility and cytoskeletal structure; TR: Cellular transport; LP: Lipid transport; Cu: Copper transmembrane transport; OT: Other; PP2: adult NEVL males.
(XLS)

Table S13 **SS1_selected-mapped-reads_nt16S.** This table shows the homology search results against DB:nt16S for SS1 selected mapped-reads. The provided information includes: mapped-read number; unique read name (name assigned to each read when the samples were pyrosequenced); read length; Blastn result against DB:nt16S; and its score and E-value, when applicable.
(XLS)

Table S14 **SS1_selected-mapped-reads_est-others.** This table shows the homology search results against DB:est-others for SS1 selected mapped-reads. The provided information includes: mapped-read number; unique read name (name assigned to each read when the samples were pyrosequenced); read length; Blastn result against DB:est-others; and its score and E-value, when applicable.
(XLS)

Table S15 **SS1_selected-mapped-reads_nr.** This table shows the homology search results against DB:nr for SS1 selected mapped-reads. The provided information includes: mapped-read number; unique read name (name assigned to each read when the samples were pyrosequenced); read length; BlastX result against DB:nr; and its score and E-value, when applicable.
(XLS)

Table S16 **SS1_selected-mapped-reads_uniprotKB.** This table shows the homology search results against DB:uniprotKB for SS1 selected mapped-reads. The provided information includes: mapped-read number; unique read name (name assigned to each read when the samples were pyrosequenced); read length; BlastX result against DB:uniprotKB; and its score and E-value, when applicable.
(XLS)

Table S17 **SS2_selected-mapped-reads_nt16S.** This table shows the homology search results against DB:nt16S for SS2 selected mapped-reads. The provided information includes: mapped-read number; unique read name (name assigned to each read when the samples were pyrosequenced); read length; Blastn result against DB:nt16S; and its score and E-value, when applicable.
(XLS)

Table S18 **SS2_selected-mapped-reads_est-others.** This table shows the homology search results against DB:est-others for SS2 selected mapped-reads. The provided information includes: mapped-read number; unique read name (name assigned to each read when the samples were pyrosequenced); read length; Blastn result against DB:est-others; and its score and E-value, when applicable.
(XLS)

Table S19 **SS2_selected-mapped-reads_nr.** This table shows the homology search results against DB:nr for SS2 selected mapped-reads. The provided information includes: mapped-read number; unique read name (name assigned to each read when the samples were pyrosequenced); read length; BlastX result against DB:nr; and its score and E-value, when applicable.
(XLS)

Table S20 **SS2_selected-mapped-reads_uniprotKB.** This table shows the homology search results against DB:uniprotKB for SS2 selected mapped-reads. The provided information includes: mapped-read number; unique read name (name assigned to each read when the samples were pyrosequenced); read length; BlastX result against DB:uniprotKB; and its score and E-value, when applicable.
(XLS)

Table S21 **PP1_selected-mapped-reads_nt16S.** This table shows the homology search results against DB:nt16S for PP1 selected mapped-reads. The provided information includes: mapped-read number; unique read name (name assigned to each read when the samples were pyrosequenced); read length; Blastn result against DB:nt16S; and its score and E-value, when applicable.
(XLS)

Table S22 **PP1_selected-mapped-reads_est-others.** This table shows the homology search results against DB:est-others for PP1 selected mapped-reads. The provided information includes: mapped-read number; unique read name (name assigned to each read when the samples were pyrosequenced); read length; Blastn result against DB:est-others; and its score and E-value, when applicable.
(XLS)

Table S23 **PP1_selected-mapped-reads_nr.** This table shows the homology search results against DB:nr for PP1 selected mapped-reads. The provided information includes: mapped-read number; unique read name (name assigned to each read when the samples were pyrosequenced); read length; BlastX result against DB:nr; and its score and E-value, when applicable.
(XLS)

Table S24 **PP1_selected-mapped-reads_uniprotKB.** This table shows the homology search results against DB:uniprotKB for PP1 selected mapped-reads. The provided information includes: mapped-read number; unique read name (name assigned to each read when the samples were pyrosequenced); read length; BlastX result against DB:uniprotKB; and its score and E-value, when applicable.
(XLS)

Table S25 **PP2_selected-mapped-reads_nt16S.** This table shows the homology search results against DB:nt16S for PP2 selected mapped-reads. The provided information includes: mapped-read number; unique read name (name assigned to each read when the samples were pyrosequenced); read length; Blastn result against DB:nt16S; and its score and E-value, when applicable.
(XLS)

Table S26 **PP2_selected-mapped-reads_est-others.** This table shows the homology search results against DB:est-others for PP2 selected mapped-reads. The provided information includes: mapped-read number; unique read name (name assigned to each read when the samples were pyrosequenced); read length; Blastn result against DB:est-others; and its score and E-value, when applicable.
(XLS)

Table S27 **PP2_selected-mapped-reads_nr.** This table shows the homology search results against DB:nr for PP2 selected mapped-reads. The provided information includes: mapped-read number; unique read name (name assigned to each read when the samples were pyrosequenced); read length; BlastX result against DB:nr; and its score and E-value, when applicable.
(XLS)

Table S28 **PP2_selected-mapped-reads_uniprotKB.** This table shows the homology search results against DB:uniprotKB for PP2 selected mapped-reads. The provided information includes: mapped-read number; unique read name (name assigned to each read when the samples were pyrosequenced); read length; BlastX result against DB:uniprotKB; and its score and E-value, when applicable.
(XLS)
